# Supplementary figures and images for: Mechanisms of resistance to VHL loss-induced genetic and pharmacological vulnerabilities
Source: EMBO Mol Med. 2025 Dec 19;18(2):599–619. doi: 10.1038/s44321-025-00361-w (PMC12905196; doi:10.1038/s44321-025-00361-w)

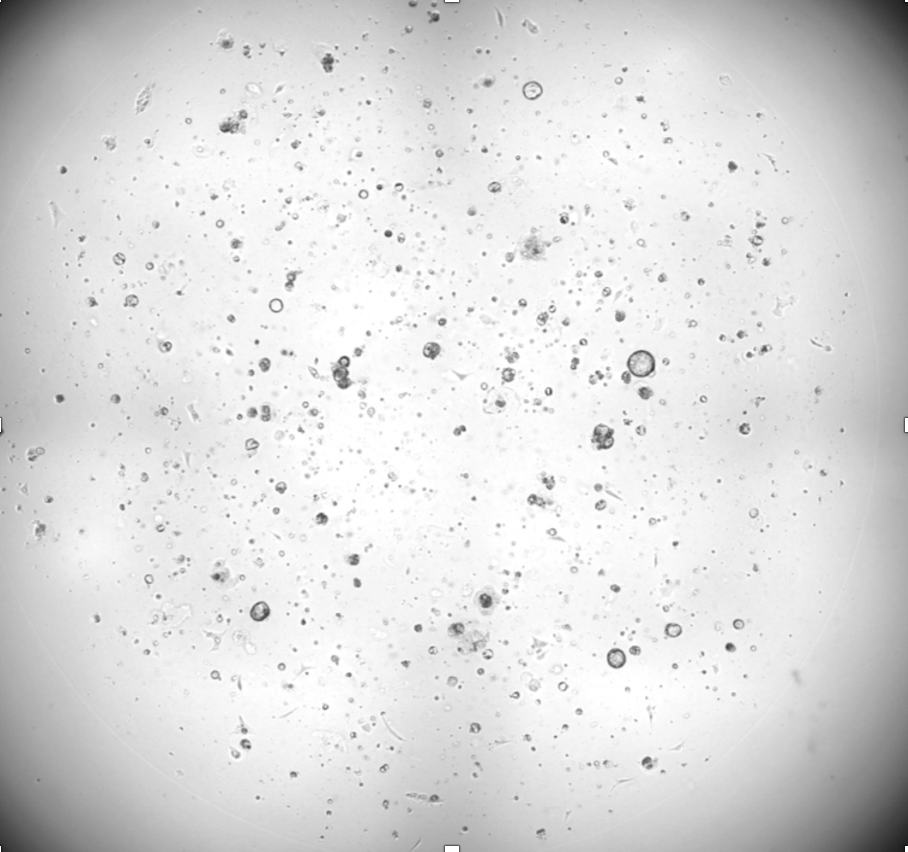

Supplement: Supplementary file 6 — Source data Fig. 1 [file 44321_2025_361_MOESM6_ESM.zip › Figure 1/1G/d0_VHL:NTC.png]

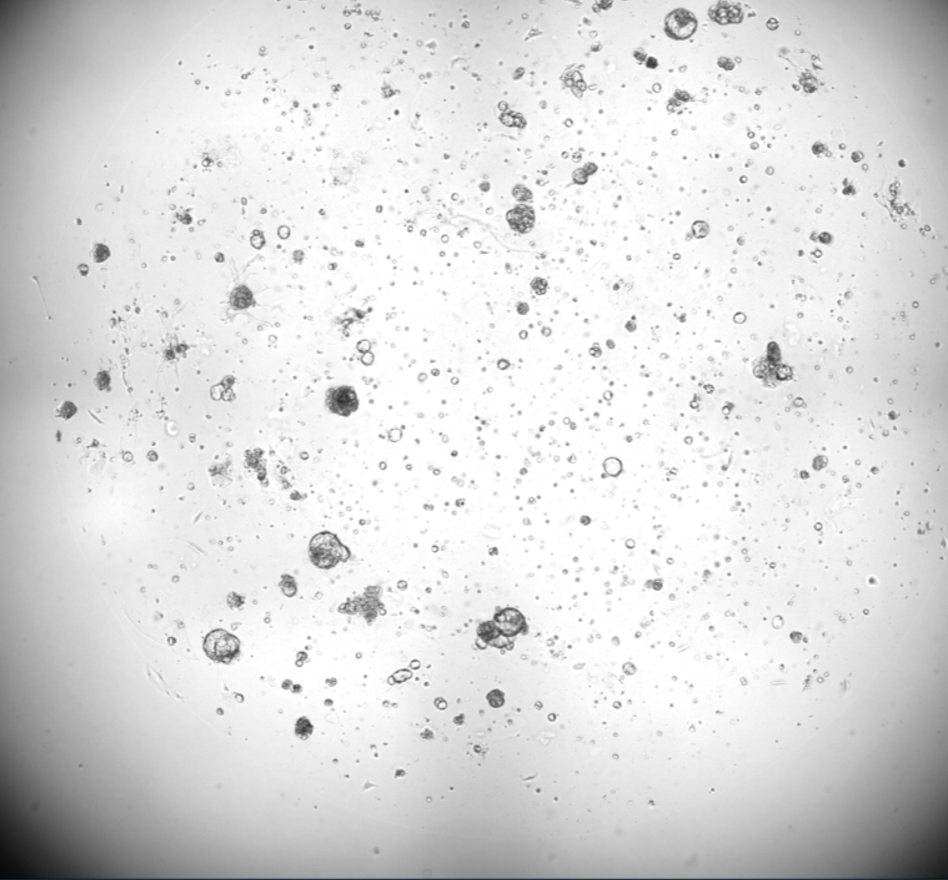

Supplement: Supplementary file 6 — Source data Fig. 1 [file 44321_2025_361_MOESM6_ESM.zip › Figure 1/1G/d0_VHL:HIF1A.png]

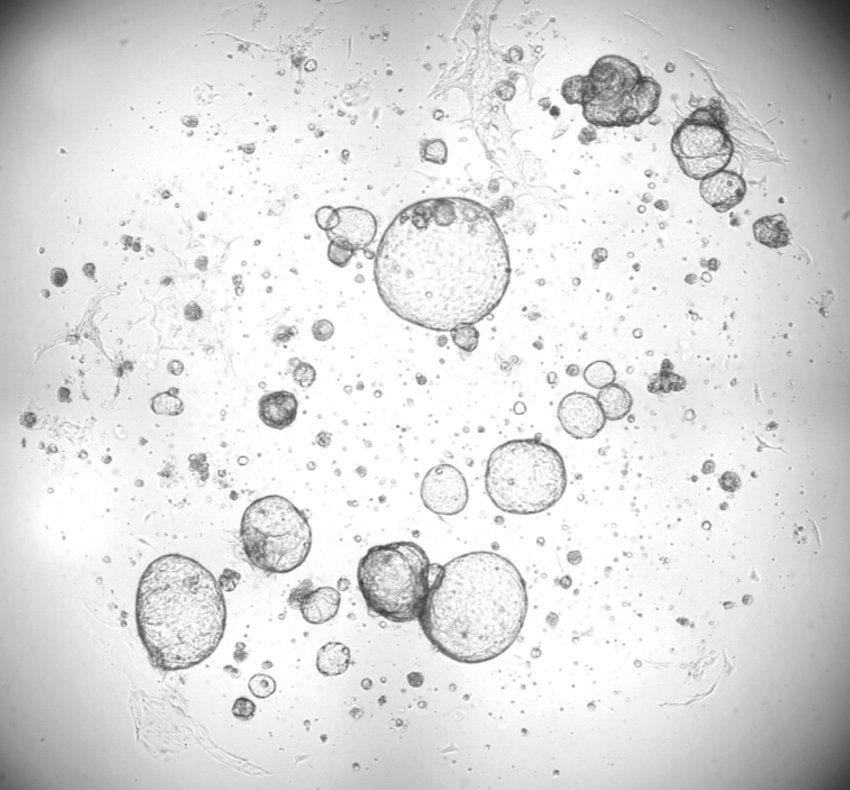

Supplement: Supplementary file 6 — Source data Fig. 1 [file 44321_2025_361_MOESM6_ESM.zip › Figure 1/1G/d12_VHL:HIF1A.png]

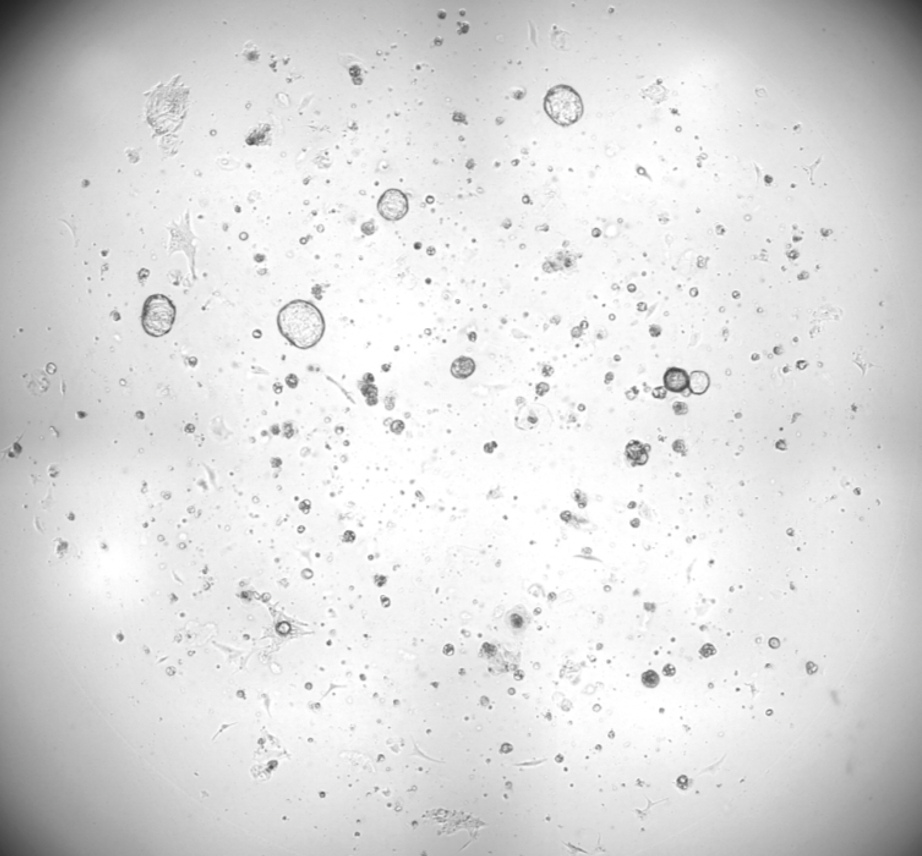

Supplement: Supplementary file 6 — Source data Fig. 1 [file 44321_2025_361_MOESM6_ESM.zip › Figure 1/1G/d12_VHL:NTC.png]

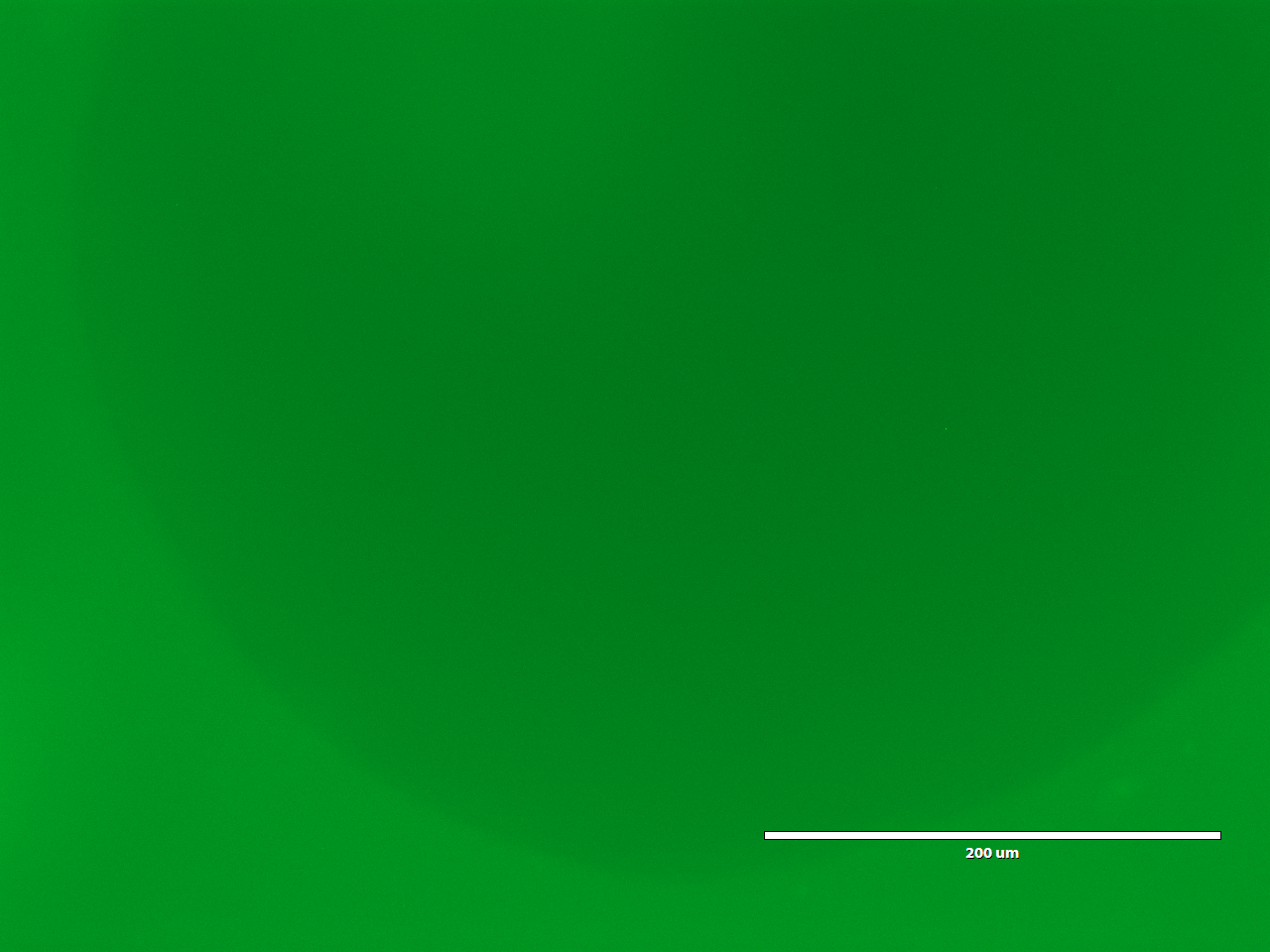

Supplement: Supplementary file 6 — Source data Fig. 1 [file 44321_2025_361_MOESM6_ESM.zip › Figure 1/1C/NTC_GFP.tif]

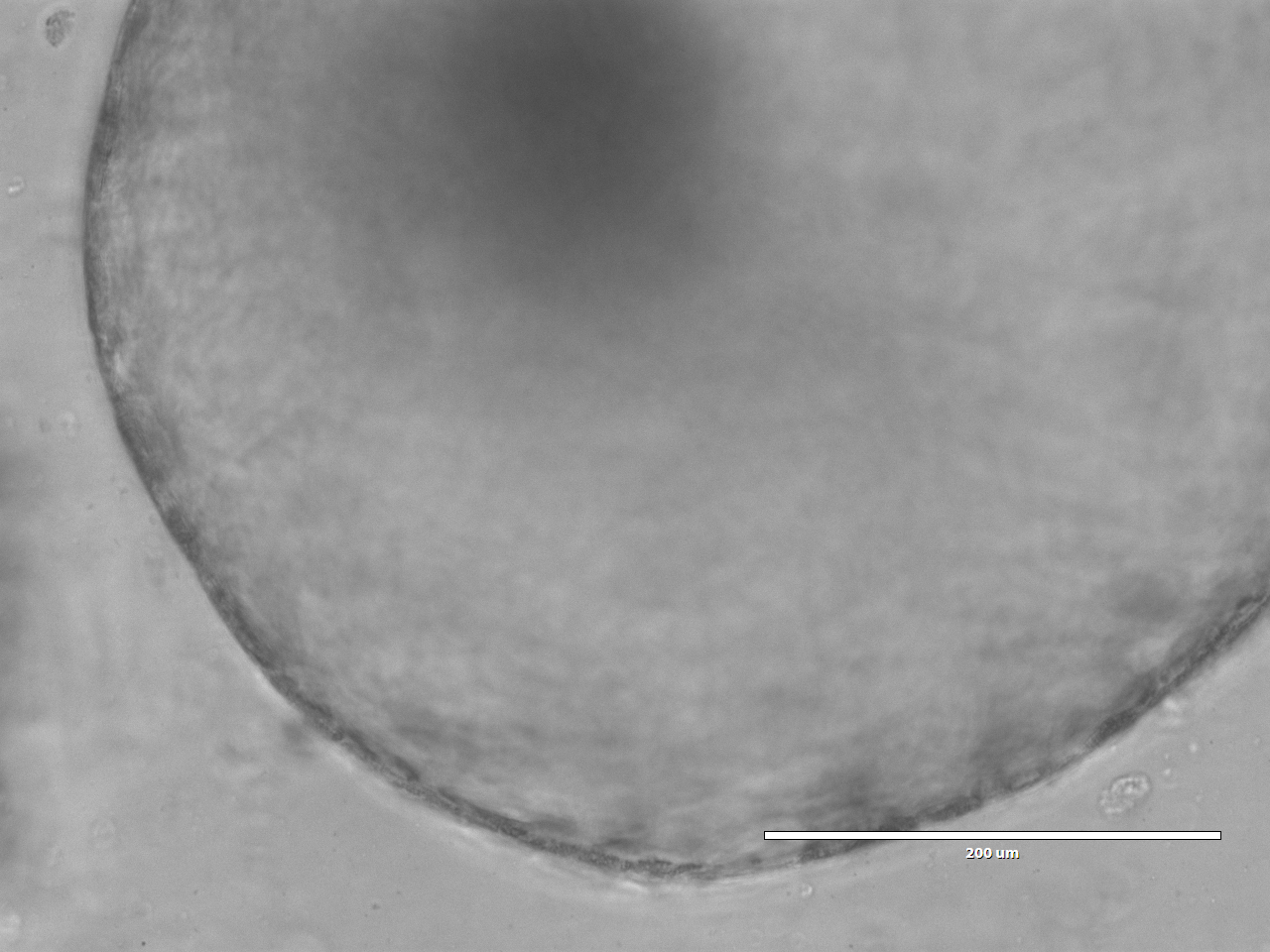

Supplement: Supplementary file 6 — Source data Fig. 1 [file 44321_2025_361_MOESM6_ESM.zip › Figure 1/1C/NTC.tif]

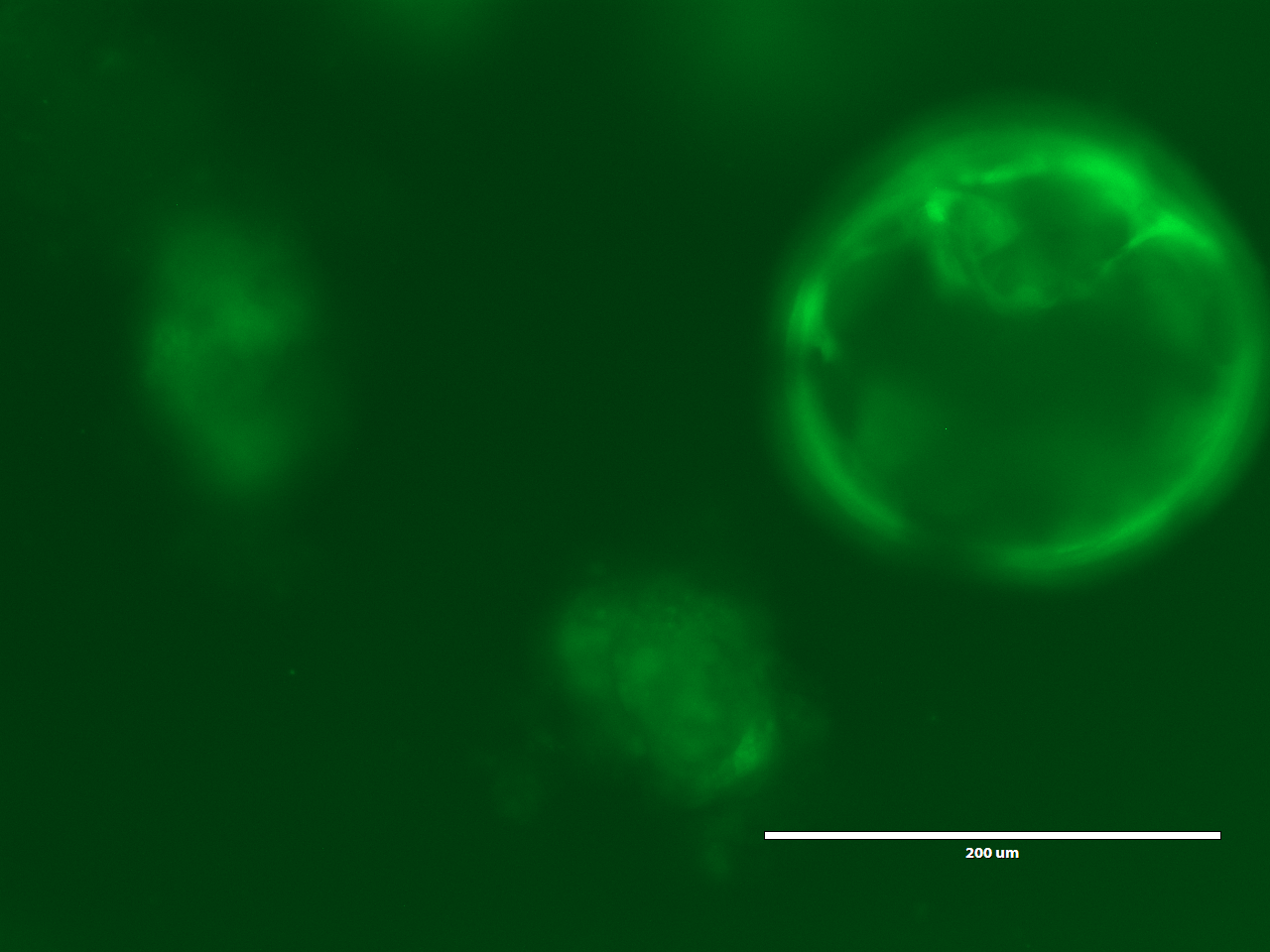

Supplement: Supplementary file 6 — Source data Fig. 1 [file 44321_2025_361_MOESM6_ESM.zip › Figure 1/1C/sg6VHL_GFP.tif]

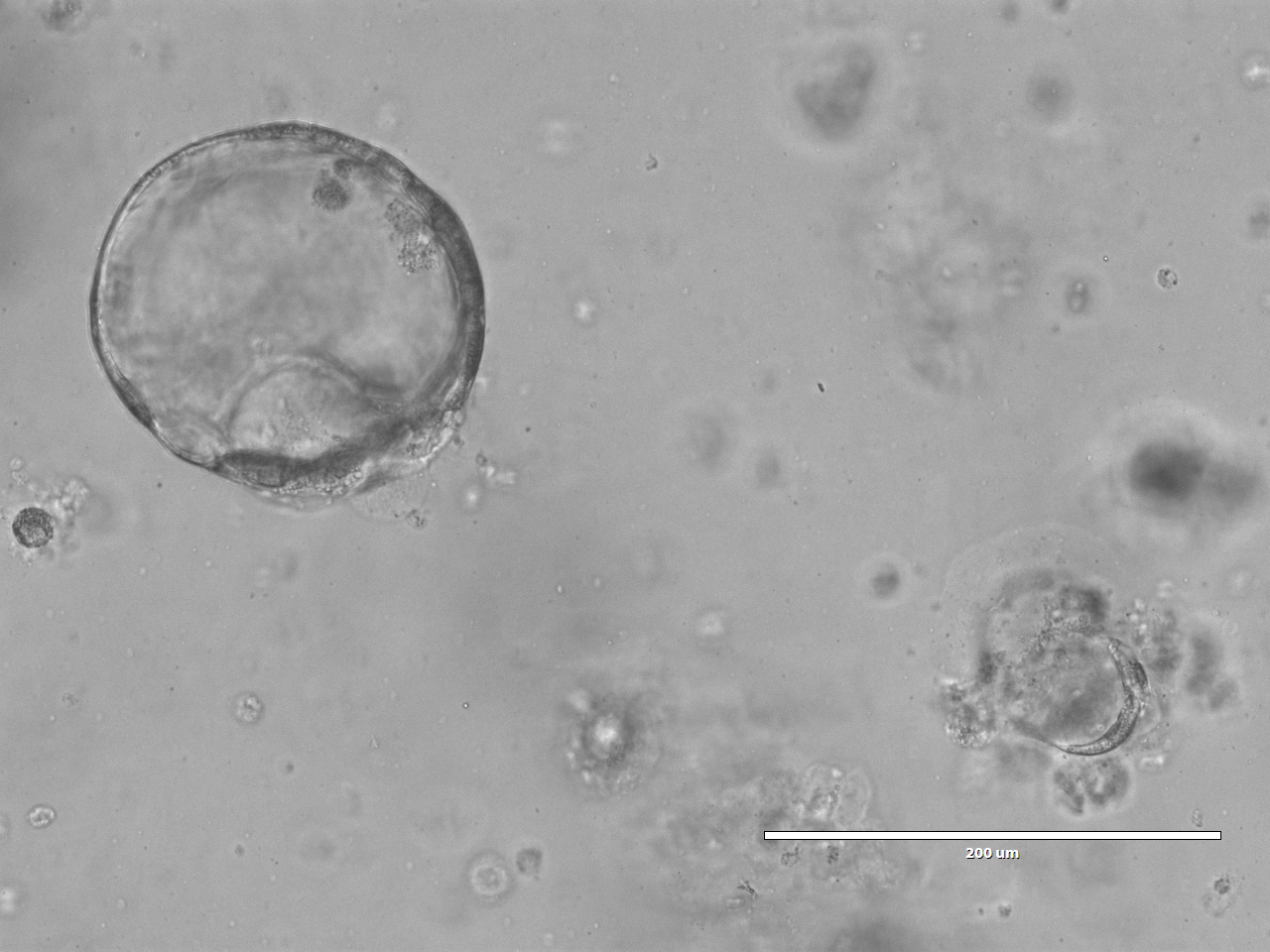

Supplement: Supplementary file 6 — Source data Fig. 1 [file 44321_2025_361_MOESM6_ESM.zip › Figure 1/1C/sg2VHL.tif]

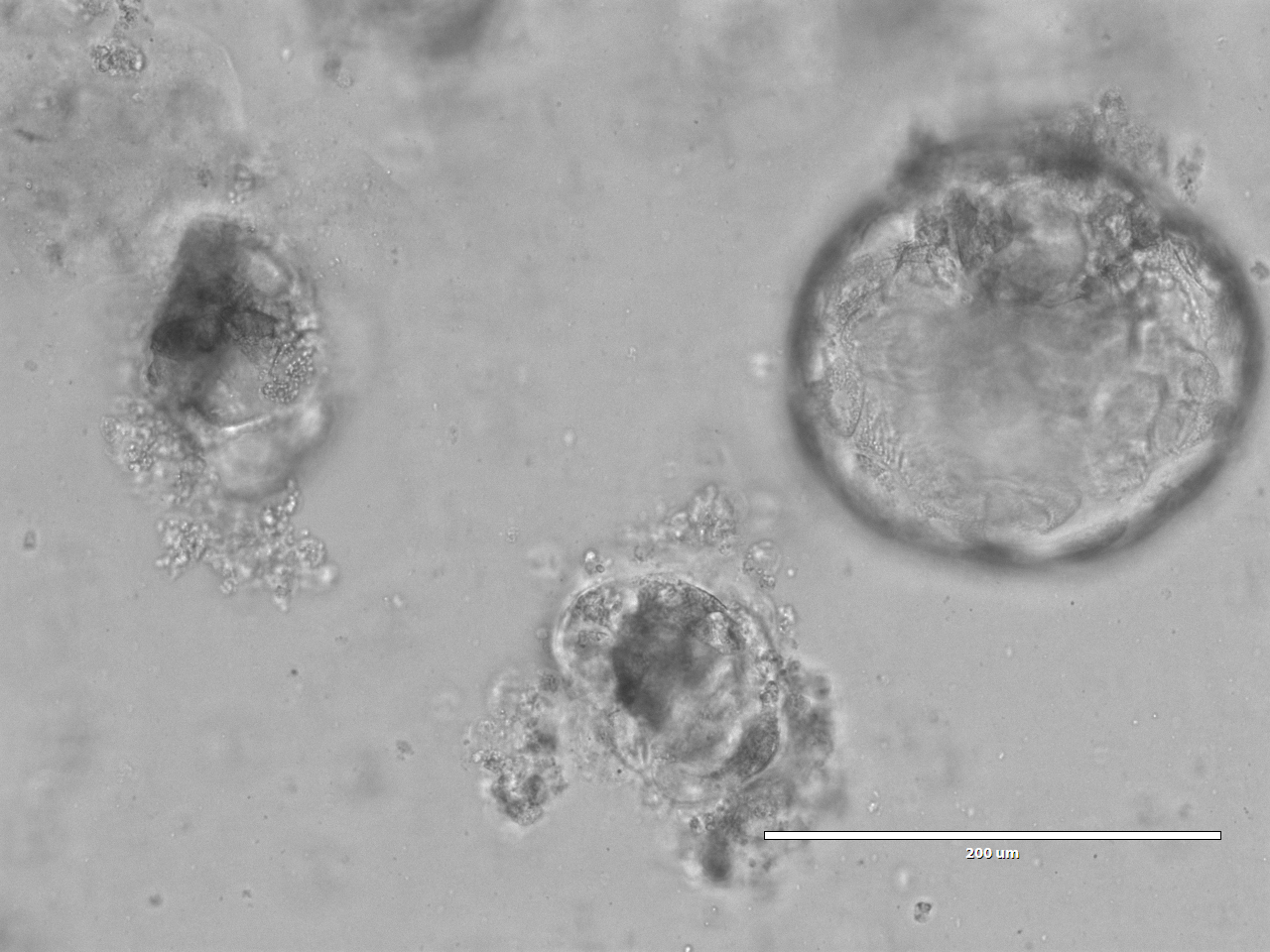

Supplement: Supplementary file 6 — Source data Fig. 1 [file 44321_2025_361_MOESM6_ESM.zip › Figure 1/1C/sg6VHL.tif]

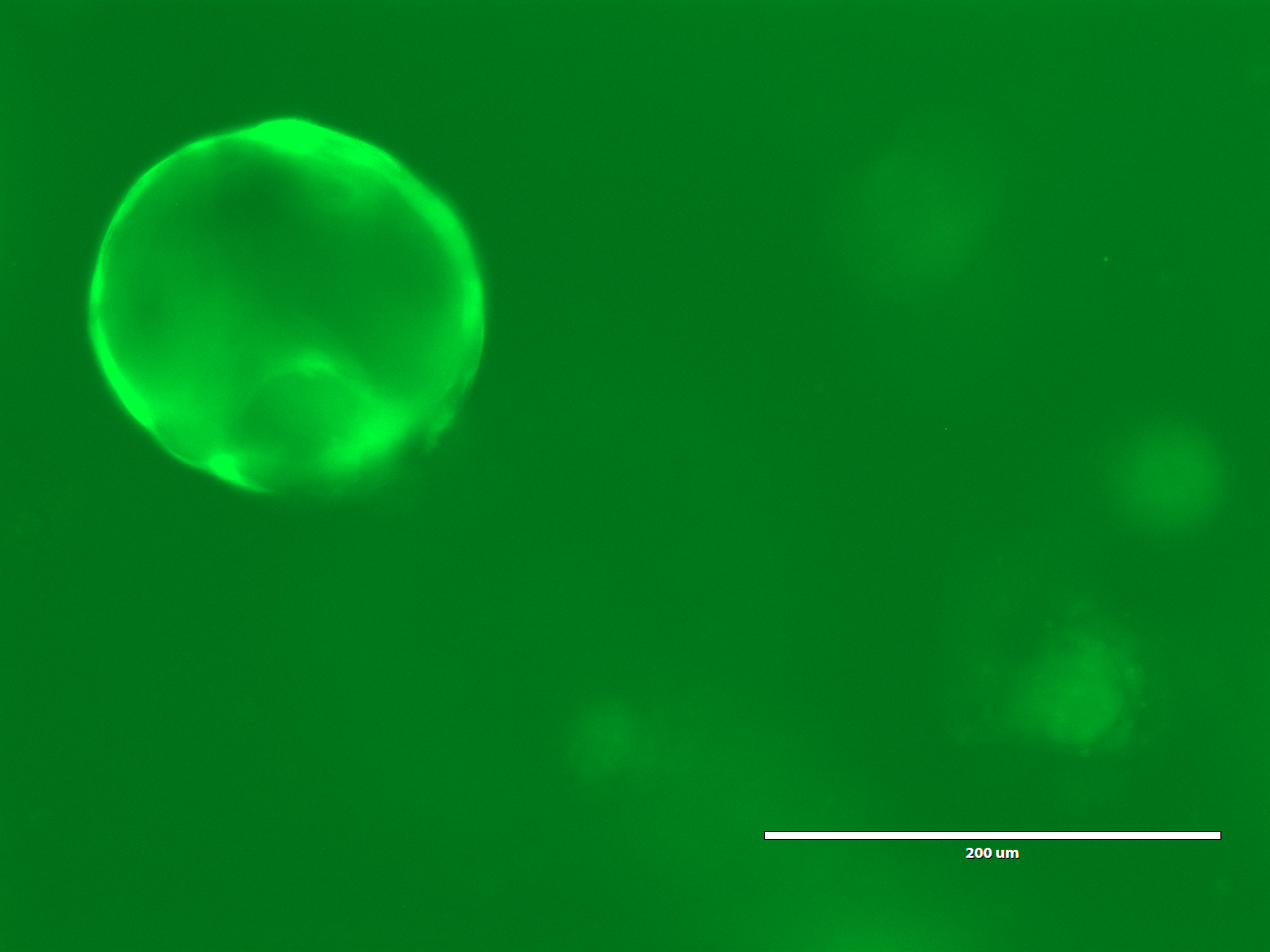

Supplement: Supplementary file 6 — Source data Fig. 1 [file 44321_2025_361_MOESM6_ESM.zip › Figure 1/1C/sg2VHL_GFP.tif]

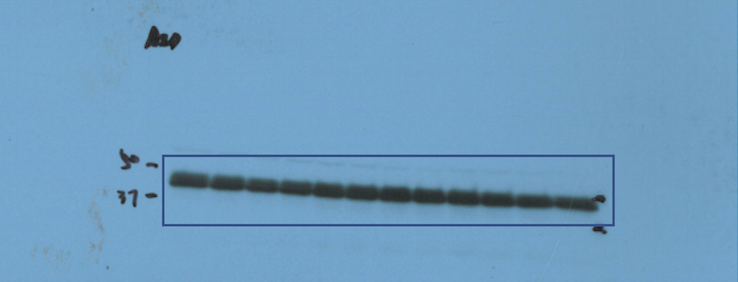

Supplement: Supplementary file 8 — Source data Fig. 3 [file 44321_2025_361_MOESM8_ESM.zip › Figure 3/3C/western_ERK_R1.png]

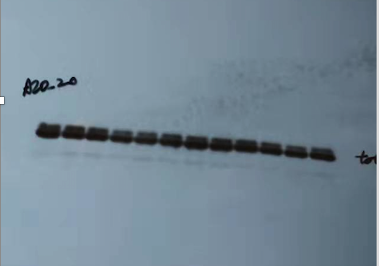

Supplement: Supplementary file 8 — Source data Fig. 3 [file 44321_2025_361_MOESM8_ESM.zip › Figure 3/3C/western_ERK_R2.png]

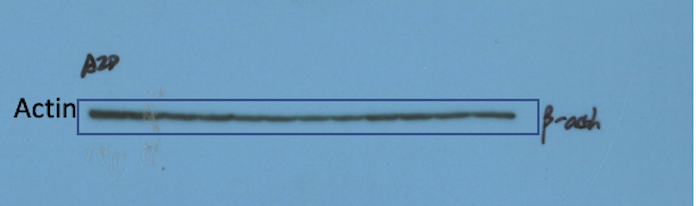

Supplement: Supplementary file 8 — Source data Fig. 3 [file 44321_2025_361_MOESM8_ESM.zip › Figure 3/3C/western_actin_R1.png]

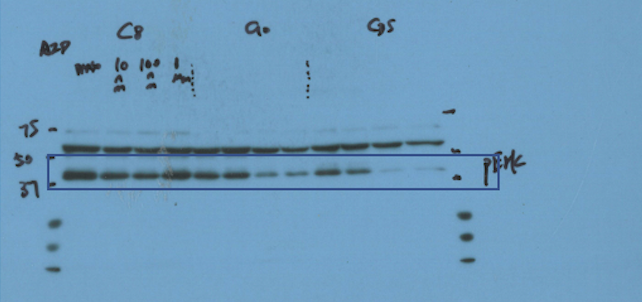

Supplement: Supplementary file 8 — Source data Fig. 3 [file 44321_2025_361_MOESM8_ESM.zip › Figure 3/3C/western_pERK_R1.png]

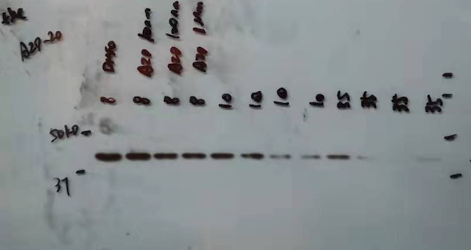

Supplement: Supplementary file 8 — Source data Fig. 3 [file 44321_2025_361_MOESM8_ESM.zip › Figure 3/3C/western_pERK_R2.png]

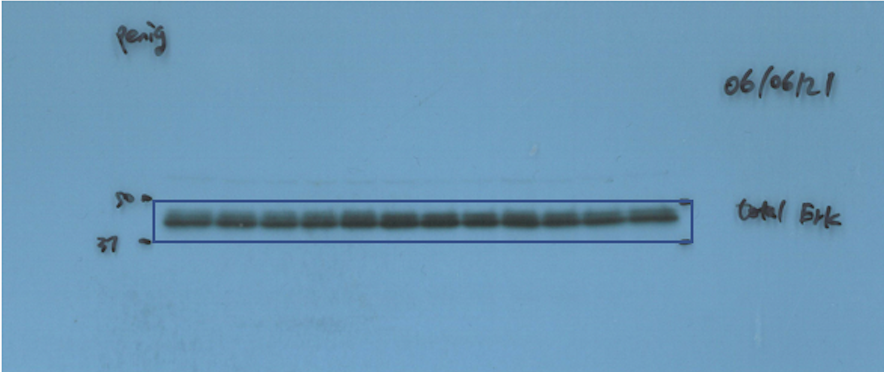

Supplement: Supplementary file 8 — Source data Fig. 3 [file 44321_2025_361_MOESM8_ESM.zip › Figure 3/3D/western_ERK_R1.png]

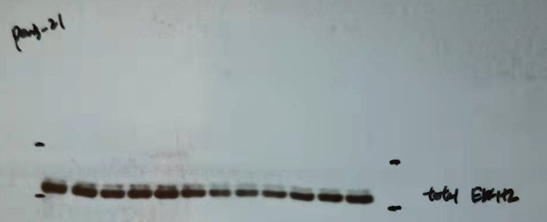

Supplement: Supplementary file 8 — Source data Fig. 3 [file 44321_2025_361_MOESM8_ESM.zip › Figure 3/3D/western_ERK_R2.png]

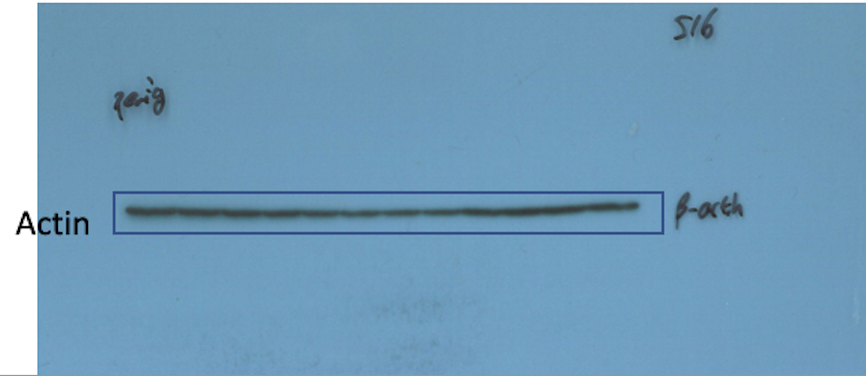

Supplement: Supplementary file 8 — Source data Fig. 3 [file 44321_2025_361_MOESM8_ESM.zip › Figure 3/3D/western_actin_R1.png]

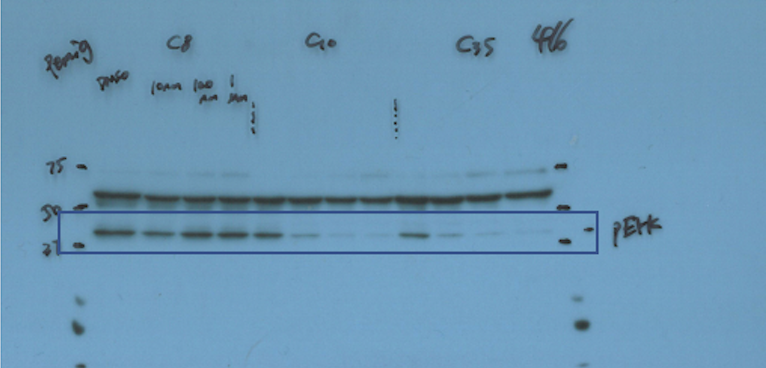

Supplement: Supplementary file 8 — Source data Fig. 3 [file 44321_2025_361_MOESM8_ESM.zip › Figure 3/3D/western_pERK_R1.png]

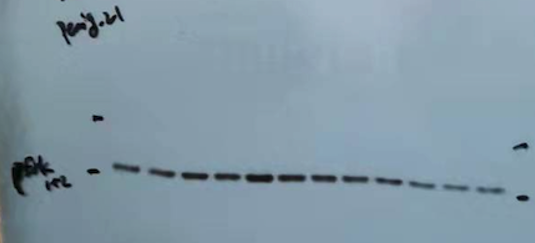

Supplement: Supplementary file 8 — Source data Fig. 3 [file 44321_2025_361_MOESM8_ESM.zip › Figure 3/3D/western_pERK_R2.png]

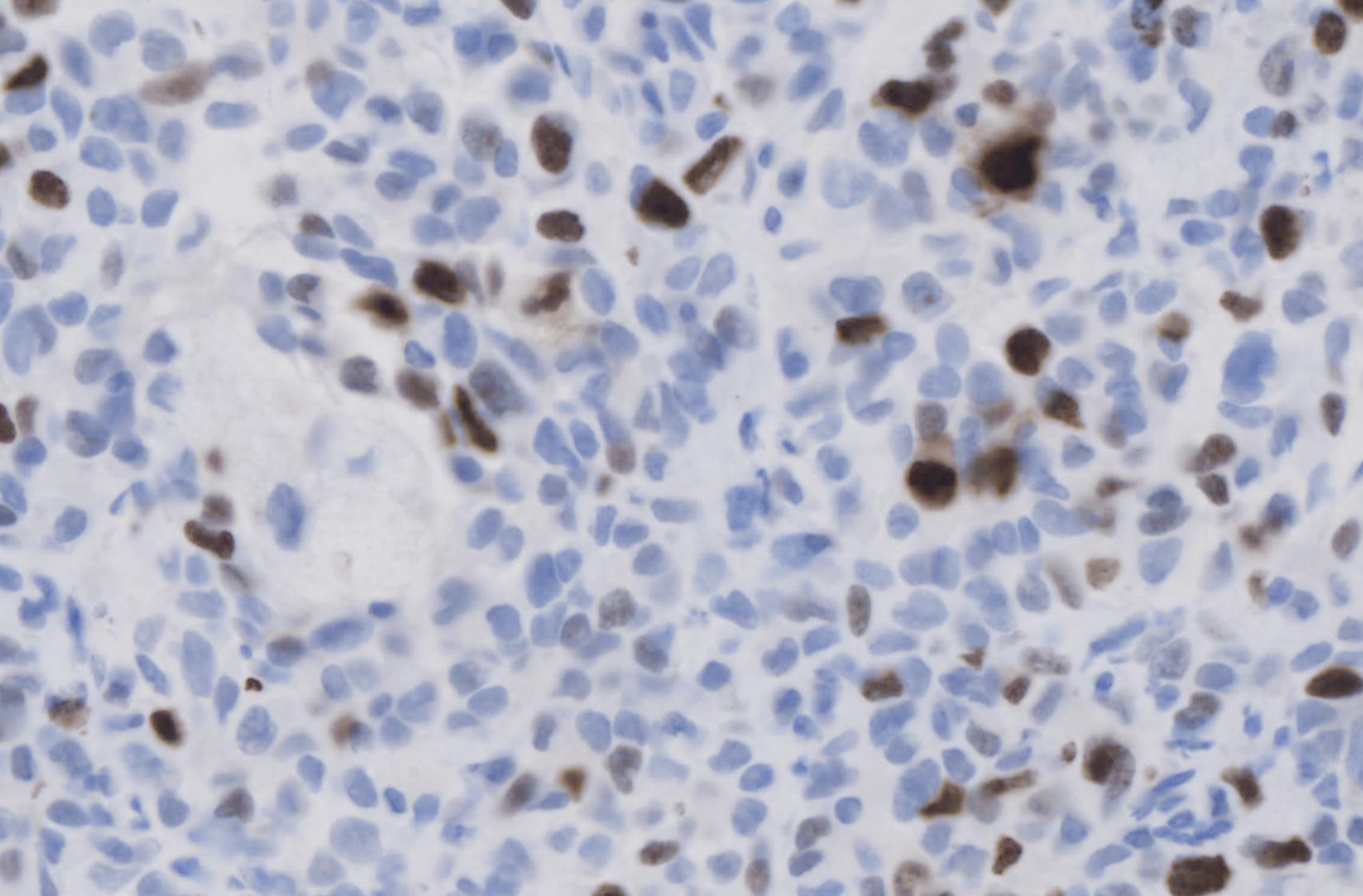

Supplement: Supplementary file 10 — Source data Fig. 5 [file 44321_2025_361_MOESM10_ESM.zip › Figure 5/5C/ARV771_Ki67.png]

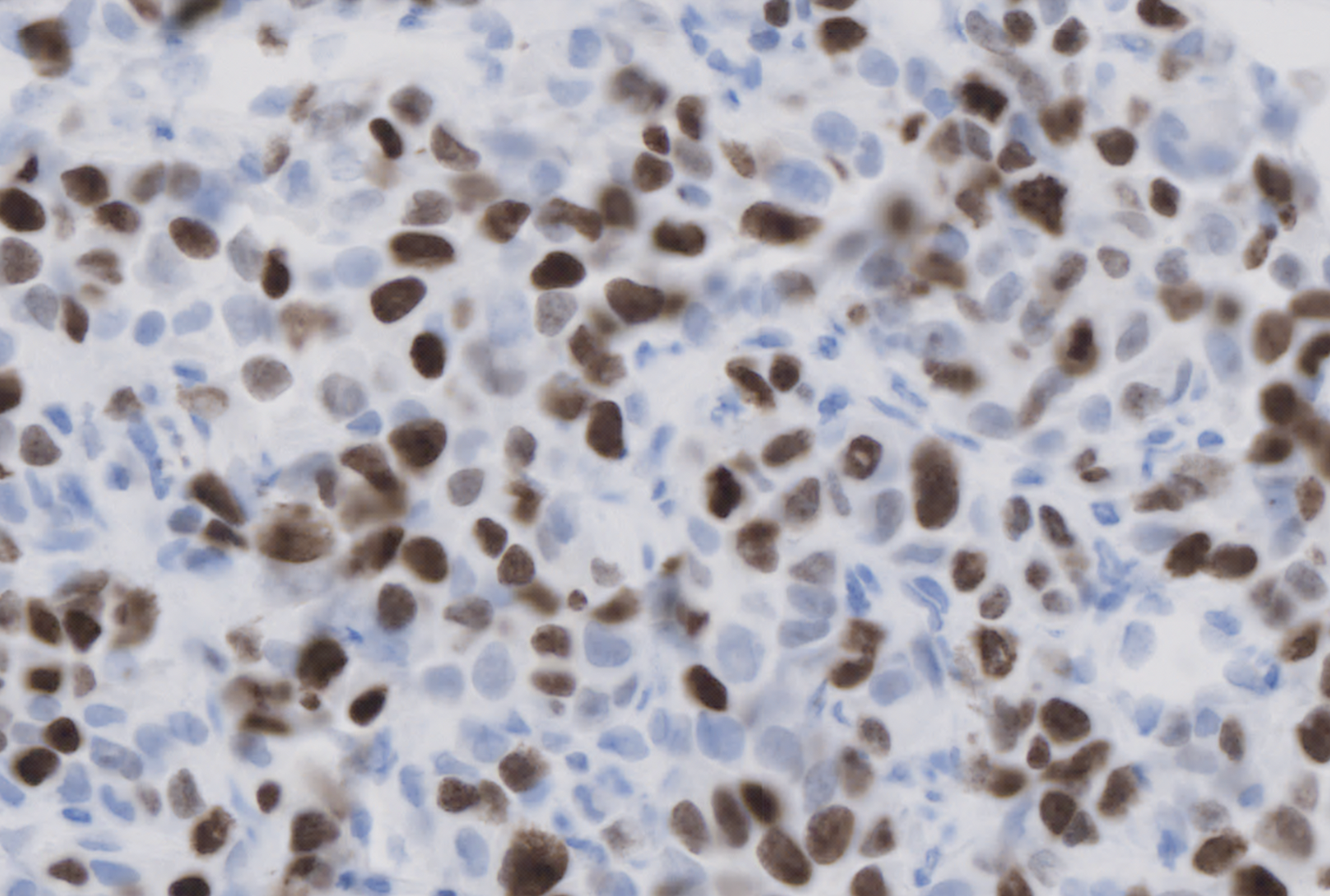

Supplement: Supplementary file 10 — Source data Fig. 5 [file 44321_2025_361_MOESM10_ESM.zip › Figure 5/5C/control_Ki67.png]

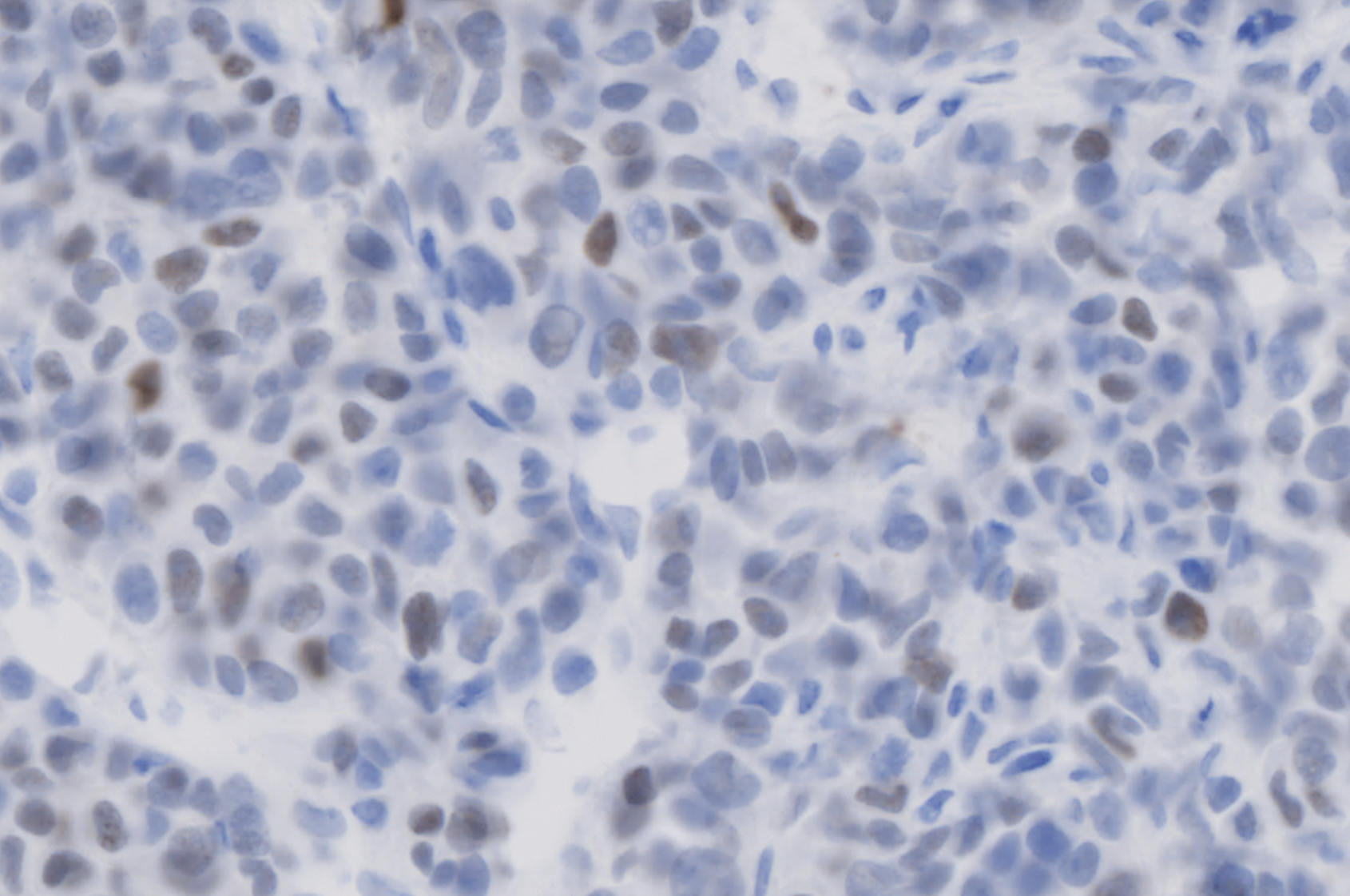

Supplement: Supplementary file 10 — Source data Fig. 5 [file 44321_2025_361_MOESM10_ESM.zip › Figure 5/5C/control_Cleaved.png]

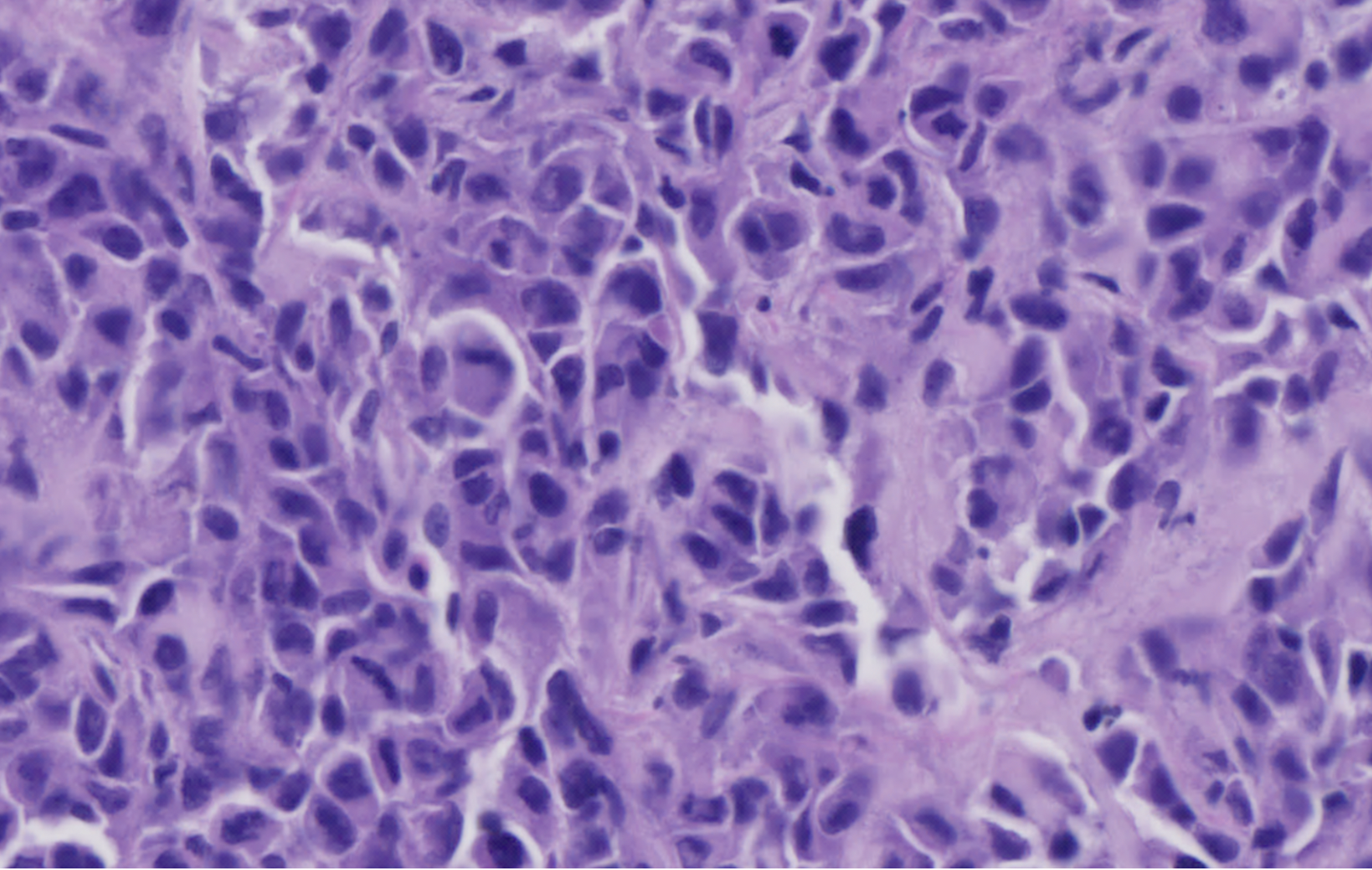

Supplement: Supplementary file 10 — Source data Fig. 5 [file 44321_2025_361_MOESM10_ESM.zip › Figure 5/5C/ARV771_HE.png]

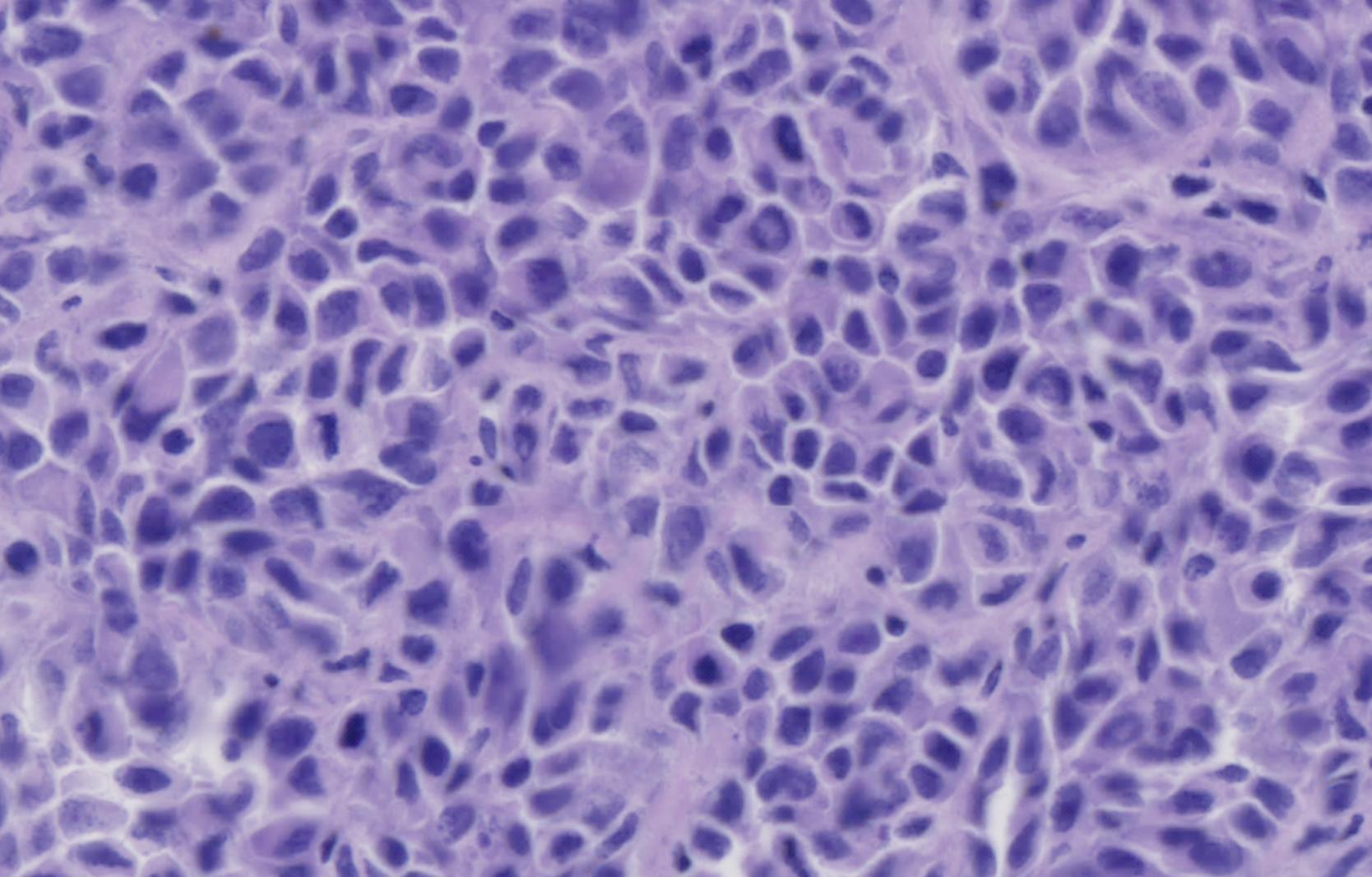

Supplement: Supplementary file 10 — Source data Fig. 5 [file 44321_2025_361_MOESM10_ESM.zip › Figure 5/5C/control_HE.png]

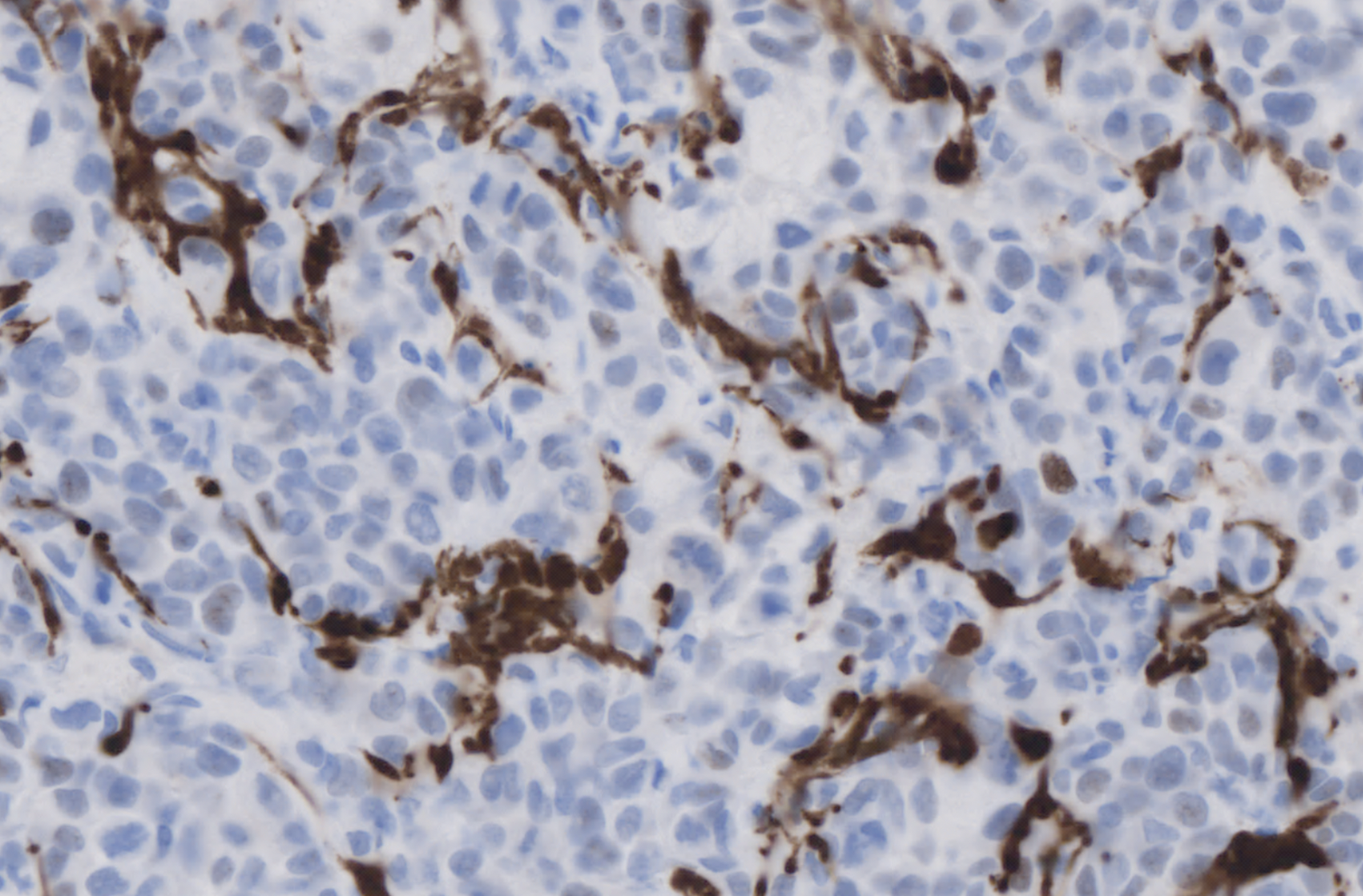

Supplement: Supplementary file 10 — Source data Fig. 5 [file 44321_2025_361_MOESM10_ESM.zip › Figure 5/5C/ARV771_Cleaved.png]

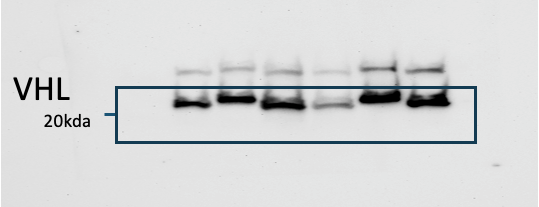

Supplement: Supplementary file 10 — Source data Fig. 5 [file 44321_2025_361_MOESM10_ESM.zip › Figure 5/5E/western_VHL.png]

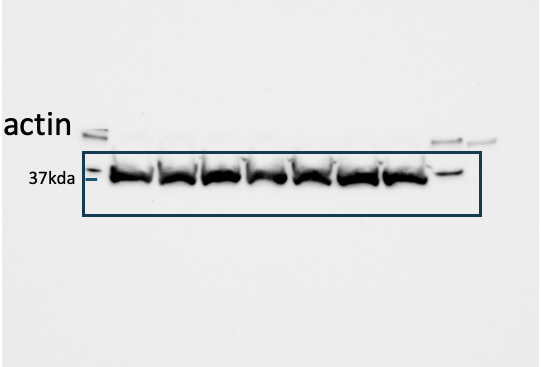

Supplement: Supplementary file 10 — Source data Fig. 5 [file 44321_2025_361_MOESM10_ESM.zip › Figure 5/5E/western_actin.png]

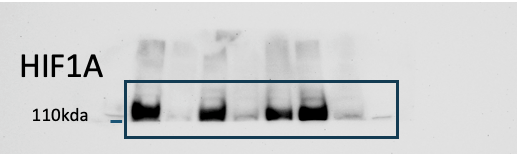

Supplement: Supplementary file 10 — Source data Fig. 5 [file 44321_2025_361_MOESM10_ESM.zip › Figure 5/5E/western_HIF1A.png]

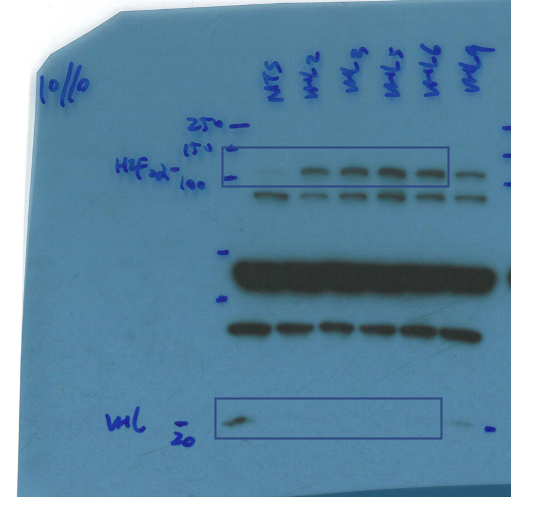

Supplement: Supplementary file 11 — Figure EV1Source Data [file 44321_2025_361_MOESM11_ESM.zip › fig.EV1/1B/Screenshot 2025-09-19 at 11.14.54.png]

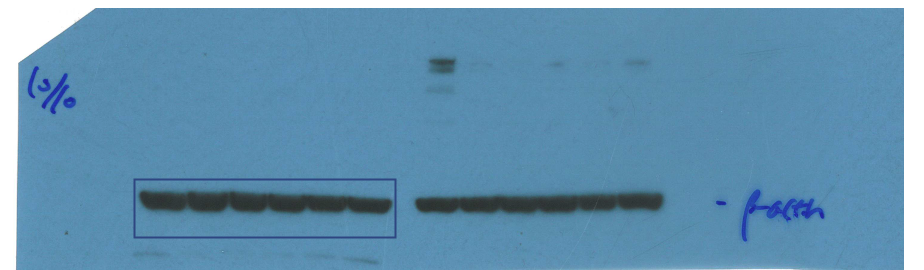

Supplement: Supplementary file 11 — Figure EV1Source Data [file 44321_2025_361_MOESM11_ESM.zip › fig.EV1/1B/Screenshot 2025-09-19 at 11.15.00.png]

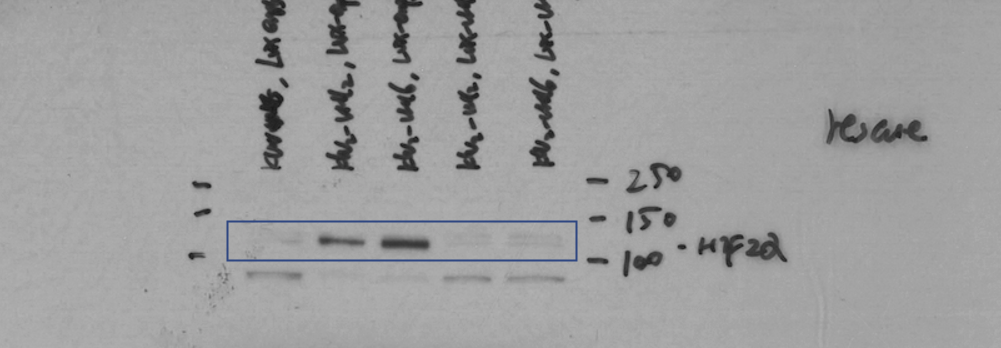

Supplement: Supplementary file 11 — Figure EV1Source Data [file 44321_2025_361_MOESM11_ESM.zip › fig.EV1/1E/Screenshot 2025-09-19 at 11.17.31.png]

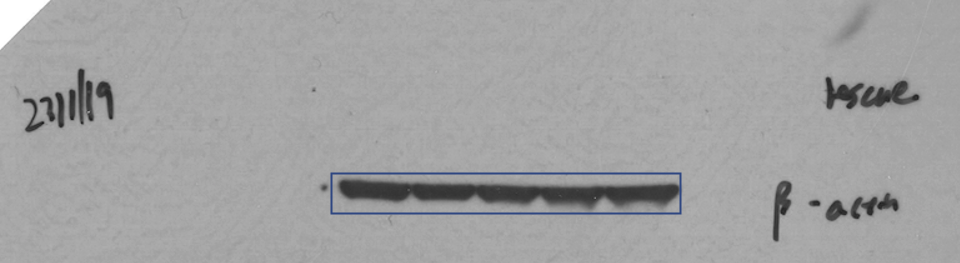

Supplement: Supplementary file 11 — Figure EV1Source Data [file 44321_2025_361_MOESM11_ESM.zip › fig.EV1/1E/Screenshot 2025-09-19 at 11.17.39.png]

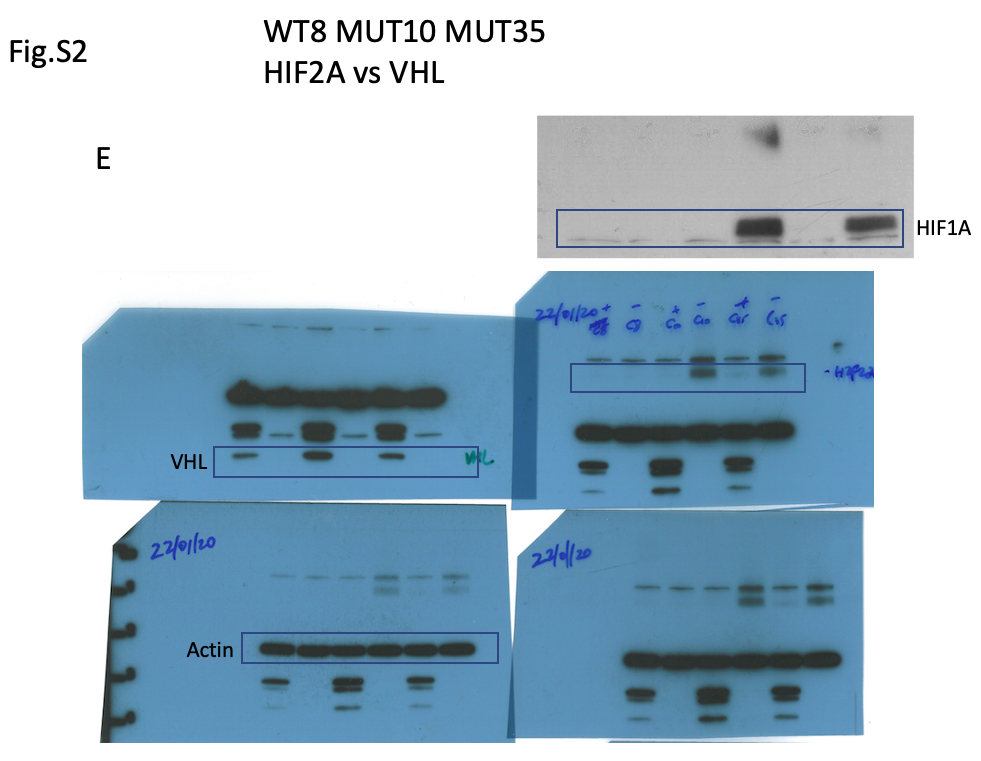

Supplement: Supplementary file 12 — Figure EV2 Source Data [file 44321_2025_361_MOESM12_ESM.zip › fig.EV2/2E/Screenshot 2025-09-19 at 11.32.59.png]

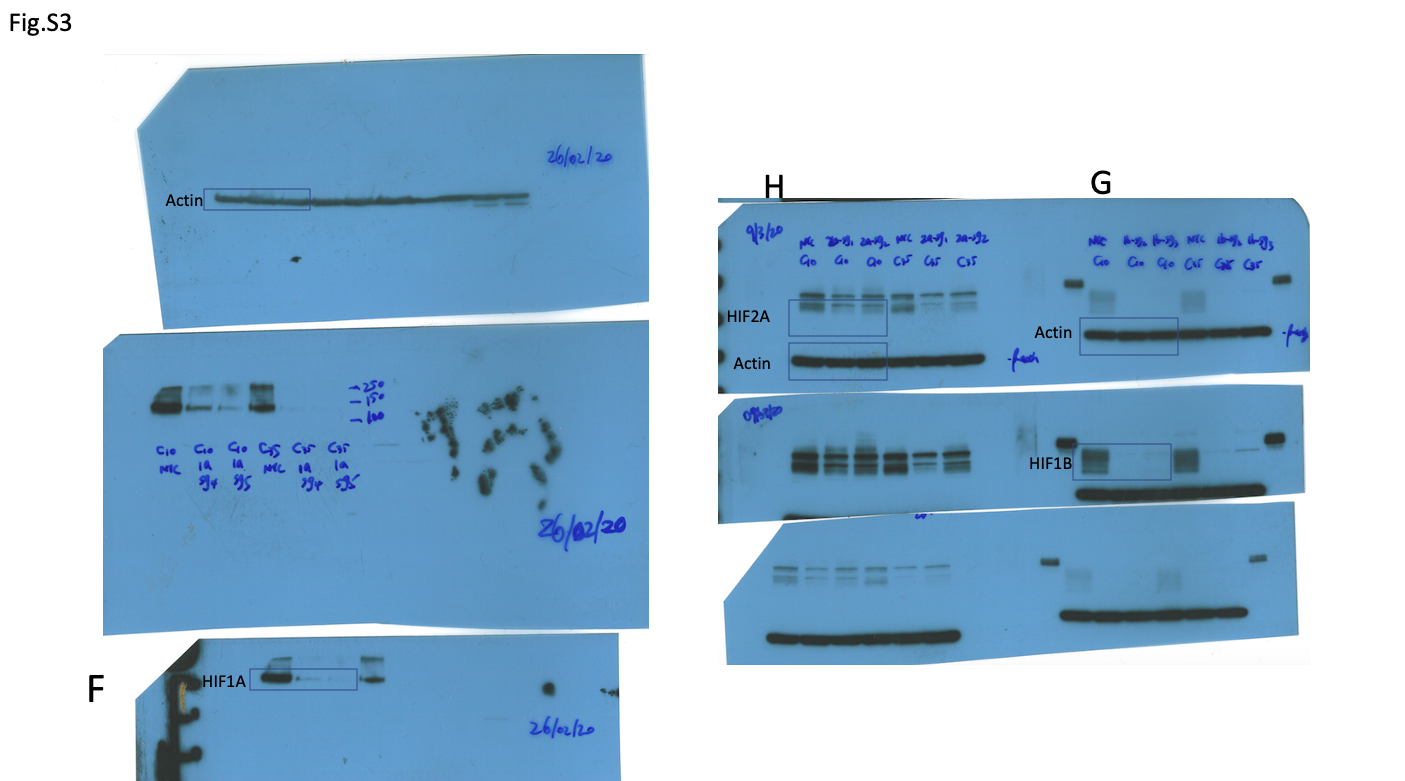

Supplement: Supplementary file 13 — Figure EV3 Source Data [file 44321_2025_361_MOESM13_ESM.zip › fig.EV3/3F-H/Screenshot 2025-09-19 at 12.12.48.png]

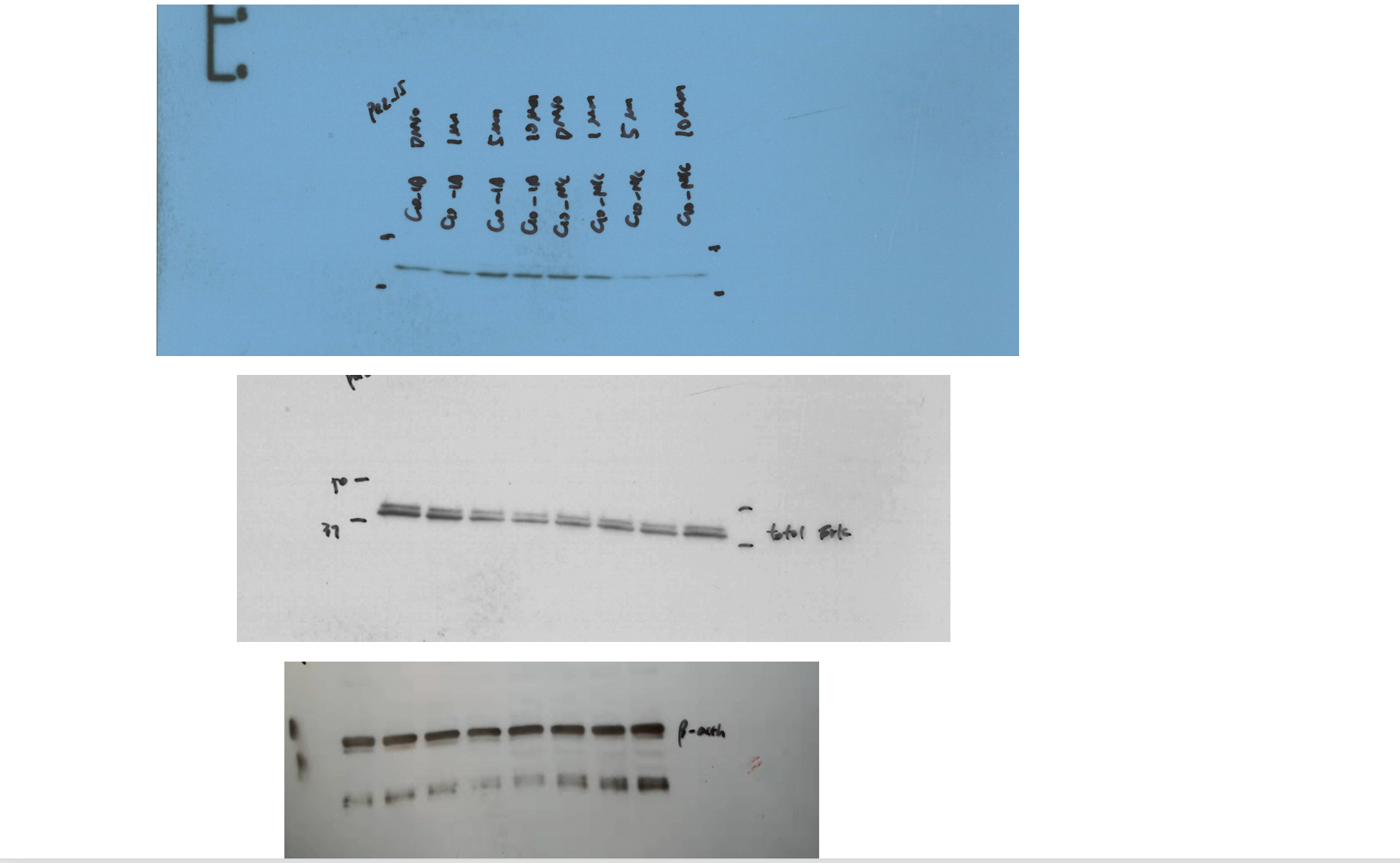

Supplement: Supplementary file 16 — Figure EV6 Source Data [file 44321_2025_361_MOESM16_ESM.zip › fig.EV6/6F/Screenshot 2025-09-20 at 23.46.25.png]

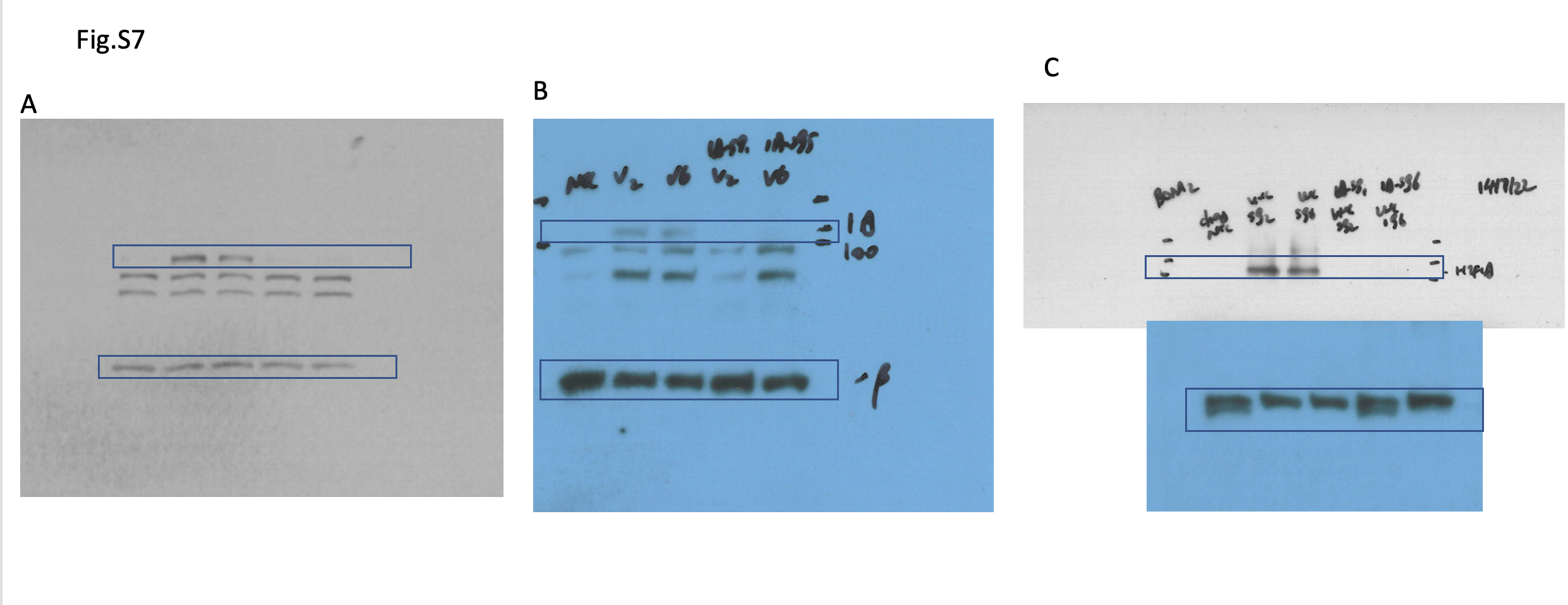

Supplement: Supplementary file 17 — Figure EV7 Source Data [file 44321_2025_361_MOESM17_ESM.zip › fig.EV7/7A-C/Screenshot 2025-09-21 at 10.30.44.png]
