# Supplementary material for: Mechanisms of resistance to VHL loss-induced genetic and pharmacological vulnerabilities
Source: EMBO Mol Med. 2025 Dec 19;18(2):599–619. doi: 10.1038/s44321-025-00361-w (PMC12905196; doi:10.1038/s44321-025-00361-w)
Supplement: Supplementary file 18 — Expanded View Figures [file 44321_2025_361_MOESM18_ESM.pdf]

Expanded View Figures

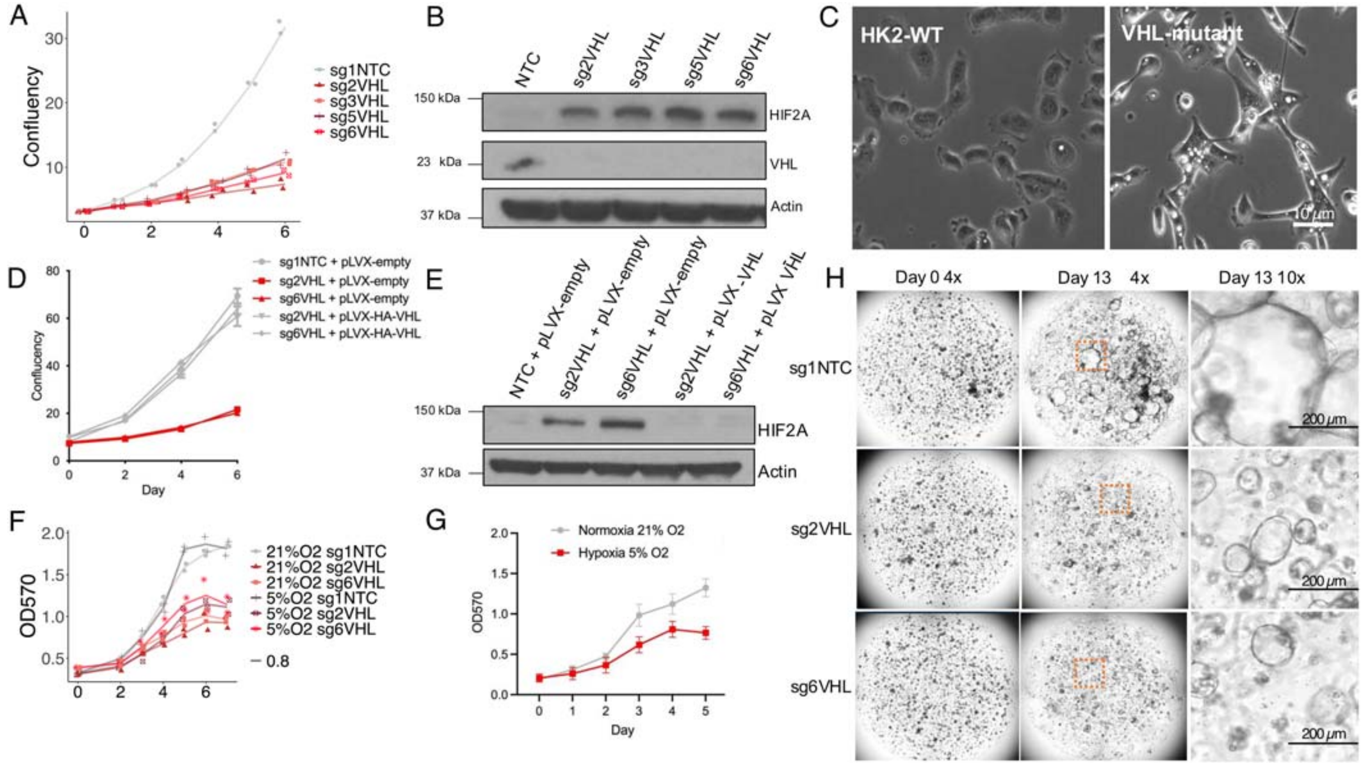

Figure EV1. VHL inactivation inhibits cell proliferation.

(**A**) Proliferation of HK2 cells with or without VHL. $N = 2$ per condition. (mean and S.E.M.). (**B**) Western blot of HIF2A, VHL and Actin on HK2 cells with and without VHL. (**C**) Morphology of HK2 cells with and without VHL. (**D**) Proliferation of VHL mutant HK2 cells with and without VHL re-introduction. $N = 3$ per condition (mean and S.E.M.). (**E**) Western blot of HIF2A on VHL mutant HK2 cells with and without VHL re-introduction. (**F**) Proliferation of HK2 cells with and without VHL under 5% or 21% O_2 culture conditions. $N = 2$ per condition (mean and S.E.M.). (**G**) Representative images of human renal epithelial organoids with and without VHL. $N = 4$ replicates per condition. (**H**) Human renal epithelial organoid proliferation under DMSO or DMOG (3 mM) treatment. Source data are available online for this figure.

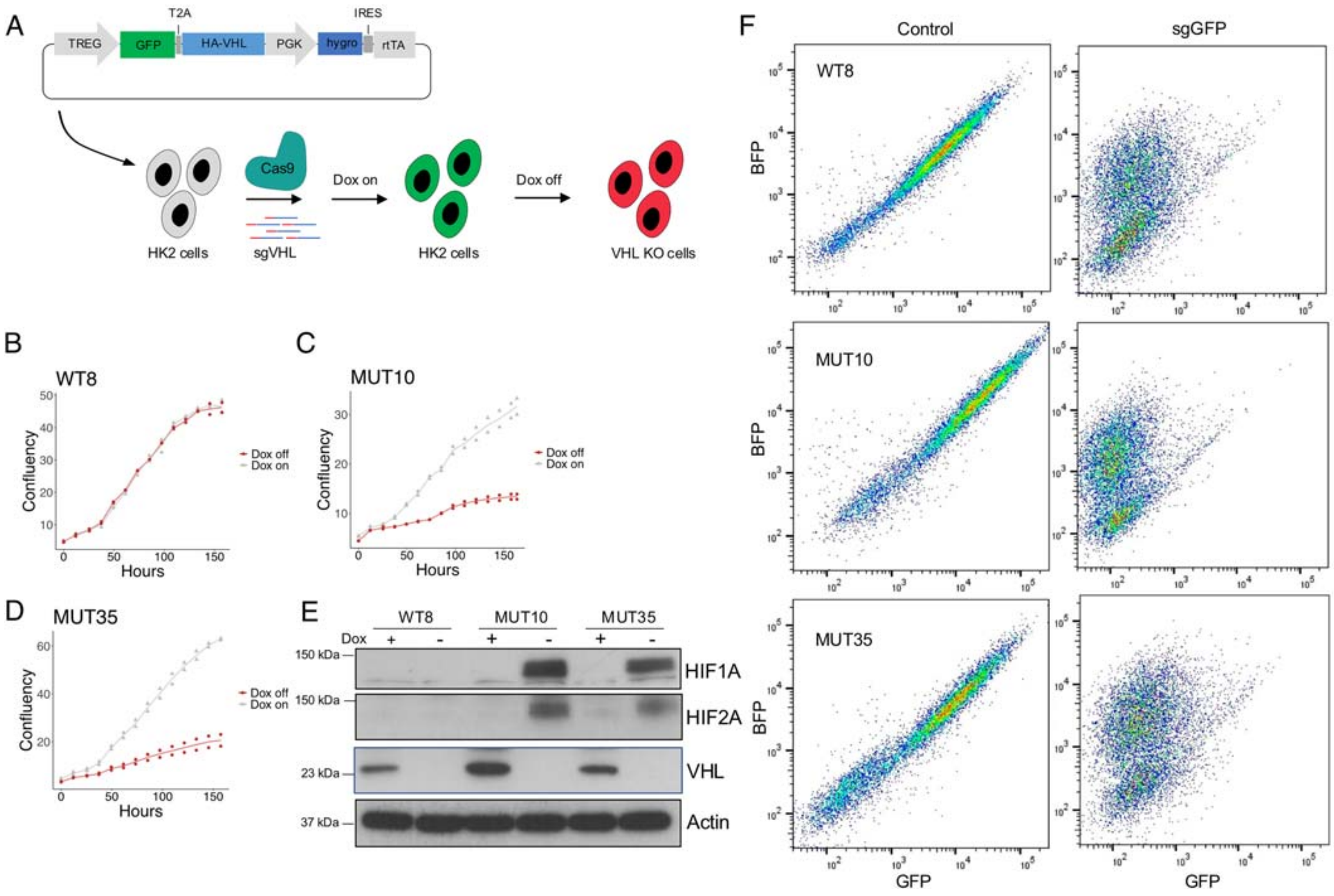

Figure EV2. Establishment of models with doxycycline-controllable VHL expression.

(**A**) Schematic of doxycycline (dox) inducible VHL re-introduction into VHL mutant (MUT10 and MUT35) and wild-type control (WT8) clones. (**B–D**) Proliferation of WT8 (**B**), MUT10 (**C**) and MUT35 (**D**) cells with and without dox. $N = 2$ replicates per condition (mean and S.E.M.). (**E**) Western blot of HIF1A, HIF2A, VHL and Actin on WT8, MUT10 and MUT35 cells with and without dox. (**F**) Cas9 editing efficiency tested on WT8, MUT10 and MUT35 cells by a reporter plasmid using fluorescence-activated cell sorting. Source data are available online for this figure.

 © The Author(s)

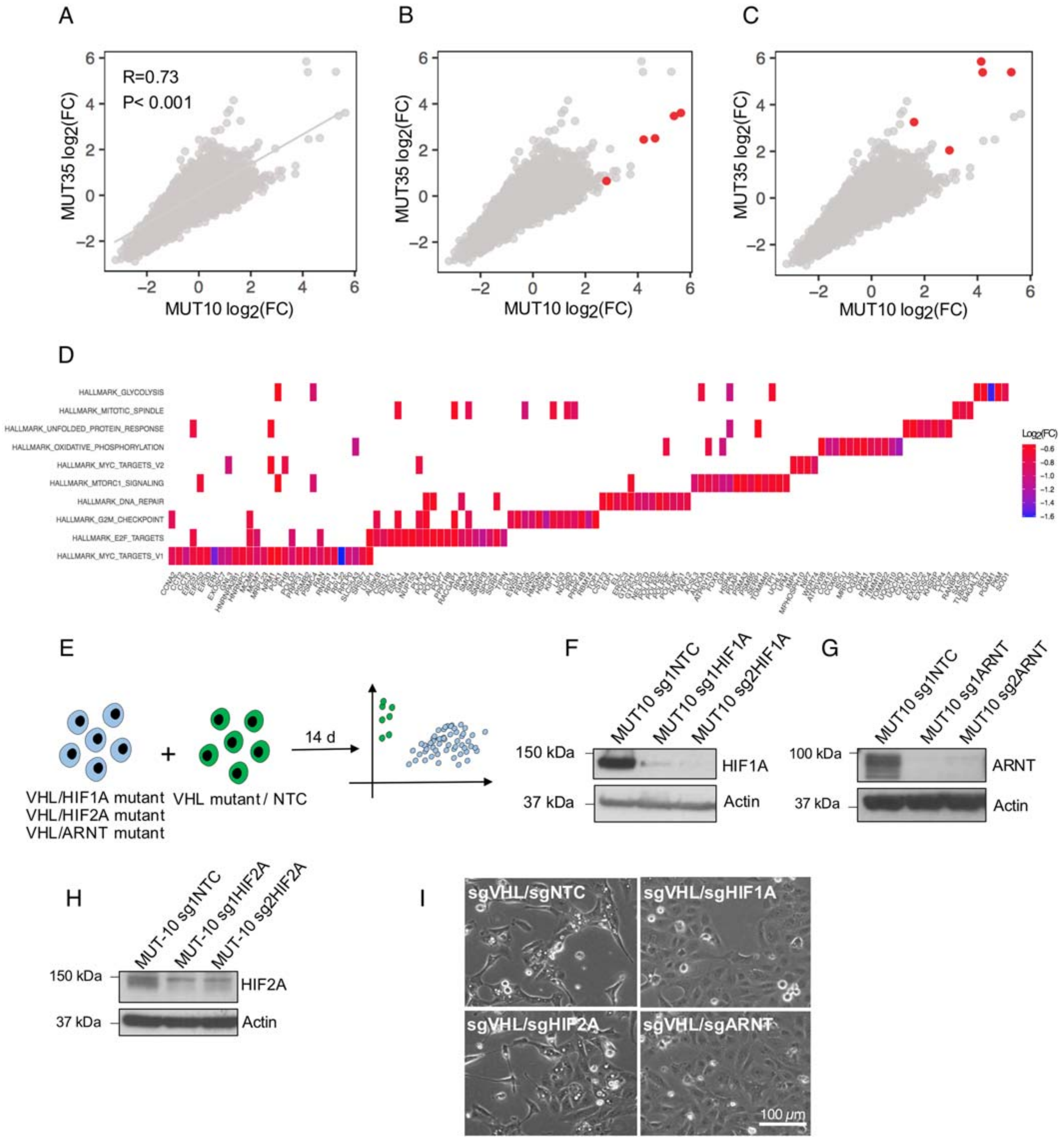

Figure EV3. HIF1A and ARNT inhibit proliferation upon VHL inactivation.

(**A**) CRISPR/Cas9-based genome wide screen data. sgRNA abundance on day 28 relative to start of the assay in VHL mutant clones MUT10 and MUT35. R, Pearson's correlation coefficient. (**B**, **C**) As in (**A**) with sgRNAs targeting genes of interest highlighted in red: HIF1A in (**B**) and ARNT in (**C**). (**D**) Pathway enrichment analysis on the top 500 genes the sgRNAs of which are depleted over time in MUT10 and MUT35 cells using the Cancer Hallmarks gene sets. (**E**) A schematic of the competitive proliferation assay. VHL-HIF1A, VHL-HIF2A and VHL-ARNT double mutant cells (BFP labelled) competed against VHL-NTC single mutant cells (GFP labelled). (**F–H**) Western blot of HIF1A, ARNT and HIF2A on MUT10 cells with and without HIF1A, ARNT or HIF2A inactivation, respectively. (**I**) Morphology of VHL-NTC, VHL-HIF1A, VHL-HIF2A and VHL-ARNT cells. Source data are available online for this figure.

© The Author(s)

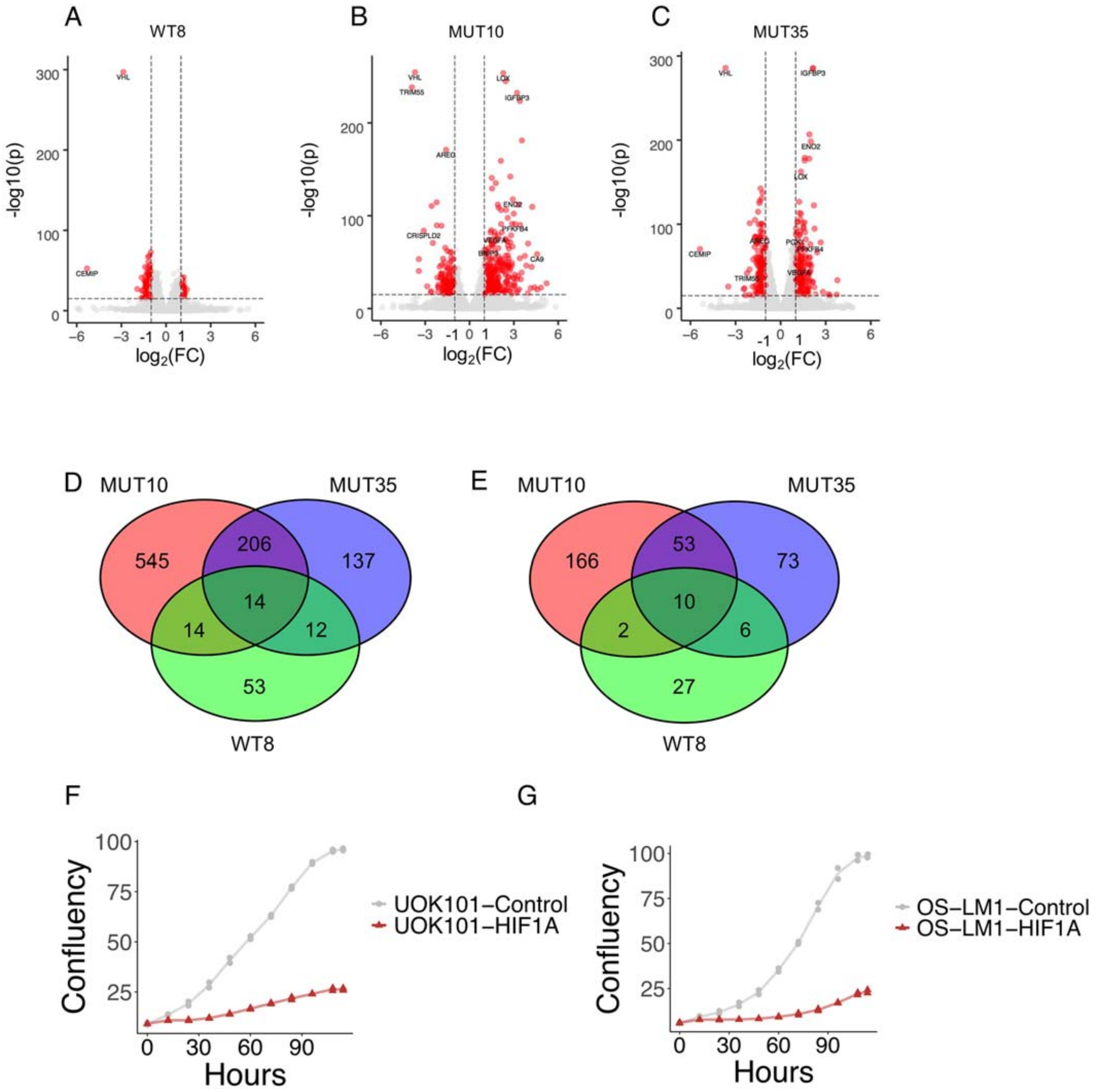

Figure EV4. HIF1A inhibits mitochondrial function.

(**A–C**) Differential gene expression analysis by RNA-seq. WT8, MUT10 and MUT35 cells, dox off compared to dox on. Adjusted *P* value and fold change determined by DESeq2, using the Wald test to test the significance. $N = 4$ replicates per condition. (**D**) Venn diagram of upregulated genes upon dox withdrawal for WT8, MUT10 and MUT35 cells. (**E**) Venn diagram of downregulated genes upon dox withdrawal for WT8, MUT10 and MUT35. (**F**) Proliferation of UOK101 ccRCC cells with and without HIF1A cDNA expression. $N = 2$ replicates per condition (mean and S.E.M.). (**G**) Proliferation of OS-LM1 ccRCC cells with and without HIF1A cDNA expression. $N = 2$ replicates per condition (mean and S.E.M.). Source data are available online for this figure.

 © The Author(s)

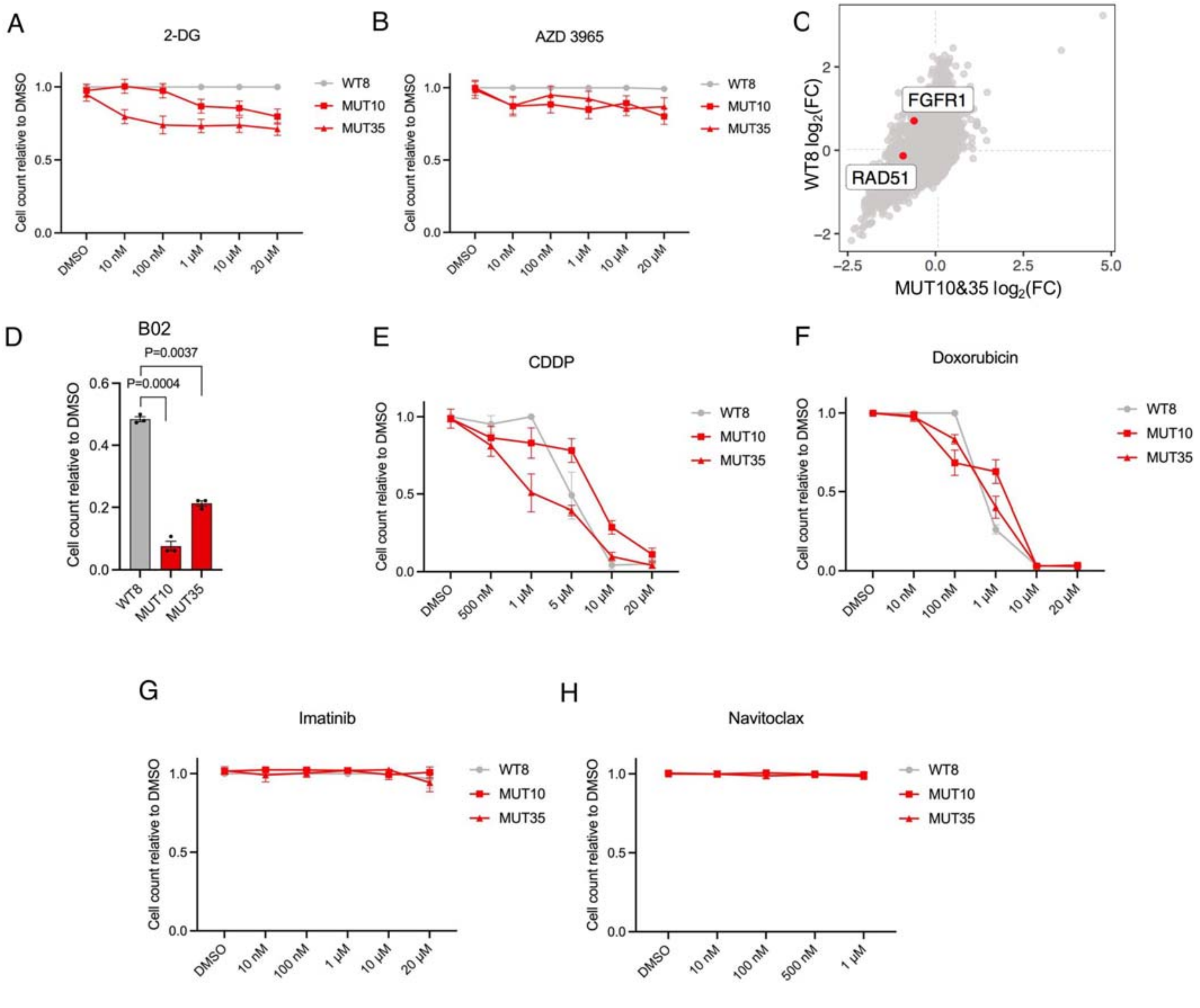

Figure EV5. *VHL* mutant cells show druggable genetic vulnerabilities.

(**A**, **B**) Cell counts on day 5 relative to DMSO control group under 2-DG (10 nM, 100 nM, 1 µM, 10 µM, 20 µM) and AZD3965 (10 nM, 100 nM, 1 µM, 10 µM, 20 µM) treatments. $N = 4$ per condition (mean and SD). (**C**) CRISPR-Cas9 screen data. FGFR1and RAD51 gene beta score distribution in in VHL WT and mutant cells. (**D**) Cell counts on day 5 relative to DMSO control group under B02 (10 µM). $N = 3$ replicates per condition (mean and SD). Paired *t* test. (**E–H**) Cell counts on day 5 relative to DMSO control group under CDDP (10 nM, 100 nM, 1 µM, 10 µM, 20 µM), Doxorubicin (10 nM, 100 nM, 1 µM, 10 µM, 20 µM), Imatinib (10 nM, 100 nM, 1 µM, 10 µM, 20 µM) and Navitoclax (10 nM, 100 nM, 1 µM, 10 µM, 20 µM). $N = 3$ replicates per condition (mean and SD). Source data are available online for this figure.

© The Author(s)

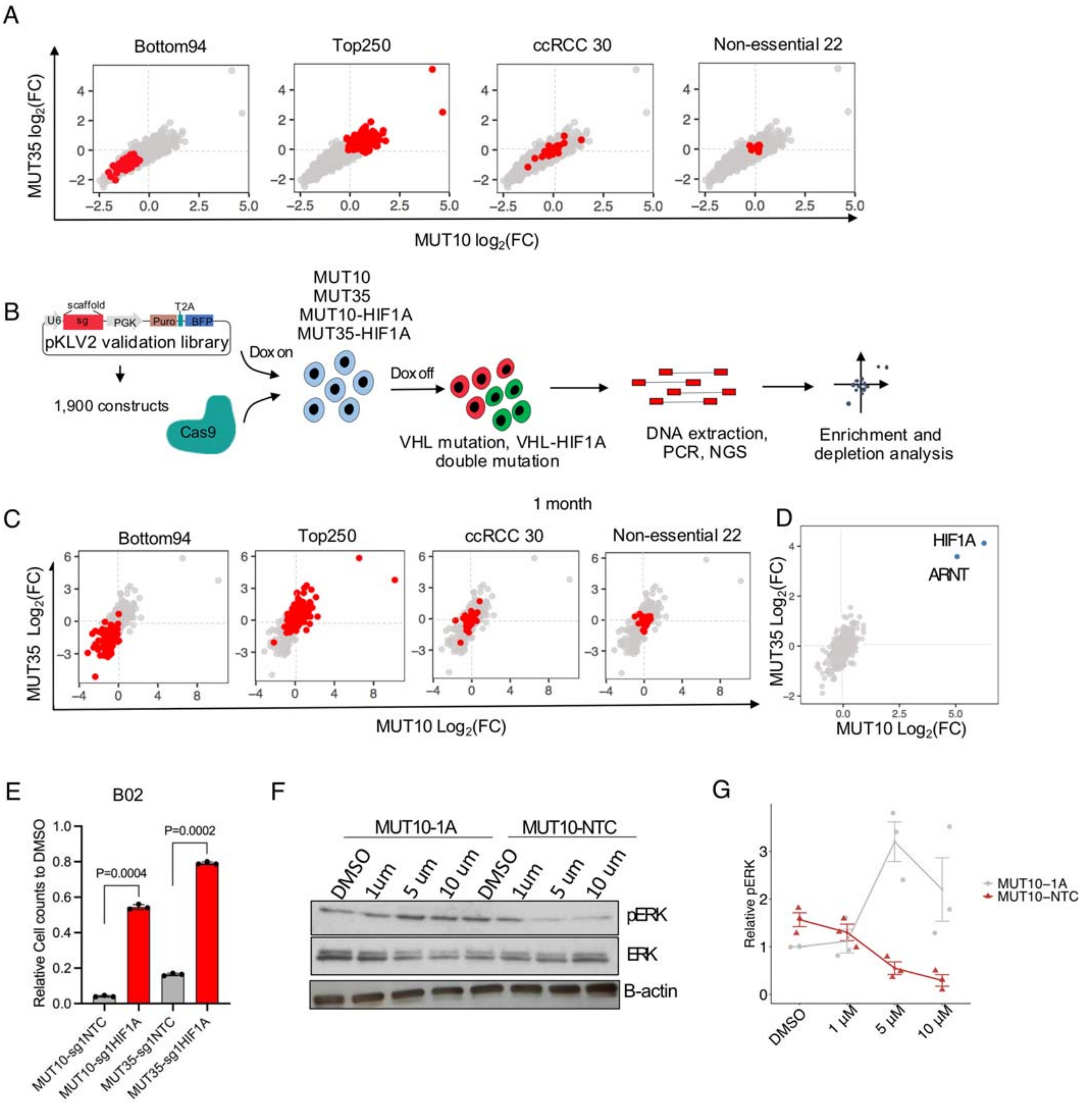

Figure EV6. CRISPR-Cas9 validation screen.

(**A**) Genes selected for the validation screen. Distribution of beta scores in the genome-wide CRISPR-Cas9 screen data set. 94 genes the constructs of which were specifically depleted in *VHL* mutant cells, 250 the constructs of which were specifically enriched in *VHL* mutant cells, 30 frequently mutated ccRCC genes and 22 non-essential control genes. (**B**) Schematic of the validation screen on MUT10, MUT35, MUT10-sgHIF1A and MUT35-sgHIF1A cells. (**C**) CRISPR-Cas9-based validation screen data. Gene level construct abundance relative to start of the assay in MUT10 and MUT35 cells. Gene sets of interest highlighted in red. (**D**) CRISPR-Cas9-based validation screen data, doxycycline withdrawal before sgRNA transduction. Gene level construct abundance relative to start of the assay in MUT10 and MUT35 cells. (**E**) Cell counts of B02 (10 μM) treated cells on day 5 relative to DMSO control group. $N = 3$ replicates per condition (mean and SD). Student's *t* test. (**F**) WB of MUT1-1A and MUT10-NTC treated with DMSO, 1 μM, 5 μM,10 μM Pazopanib for pERK, total ERK and beta-actin. (**G**) Quantification of relative pERK at different treatment concentration at indicated cell population. $N = 3$ replicates per condition (mean and S.E.M.). Source data are available online for this figure.

© The Author(s)

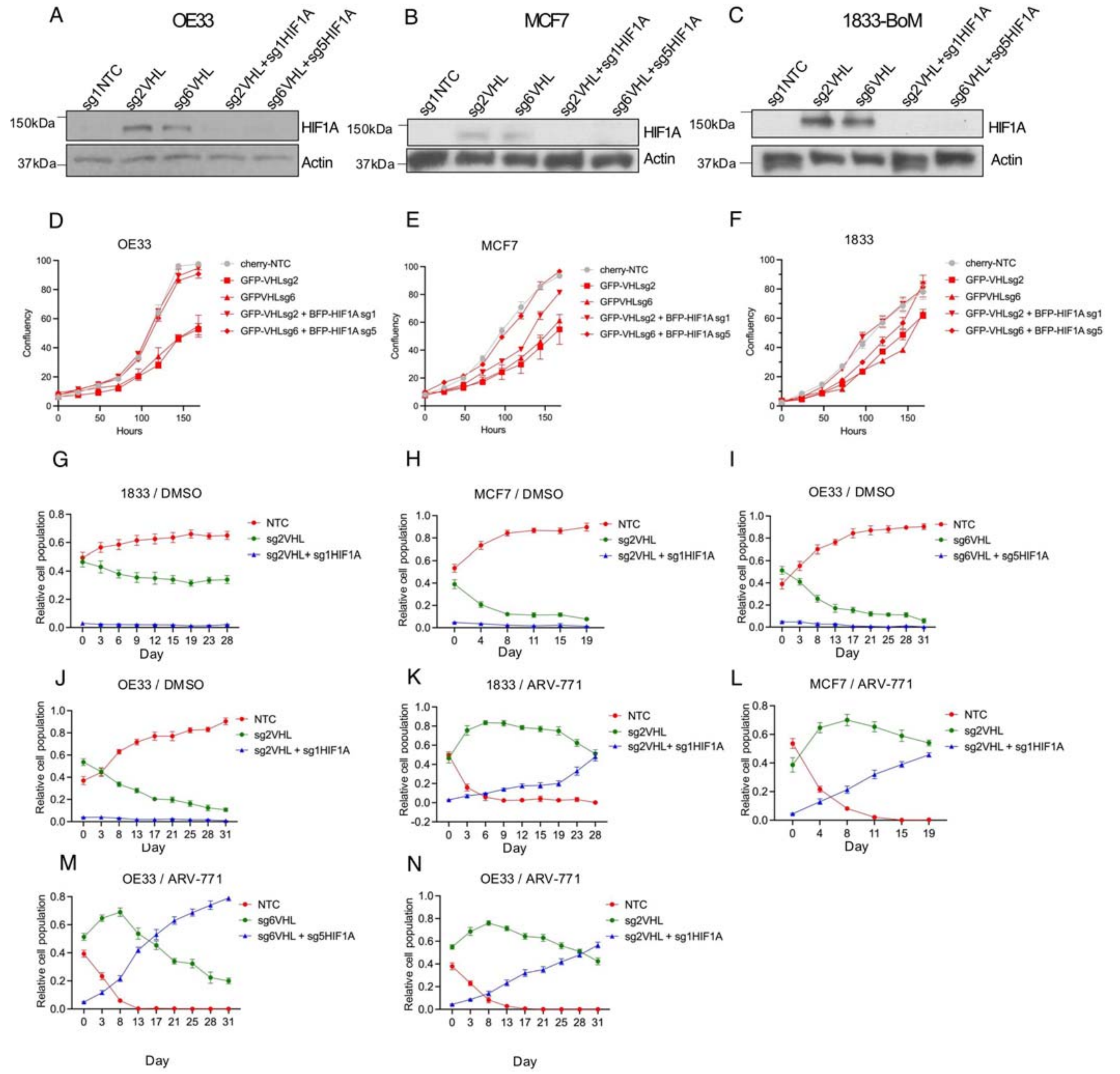

Figure EV7. HIF1A loss-mediated escape from PROTAC-induced growth inhibition in cancer.

(**A**–**C**) Western blot of HIF1A and Actin on WT (NTC), VHL mutant (VHLsg2, VHLsg6) and VHL-HIF1A double mutant (VHLsg2/HIF1Asg1, VHLsg6/HIF1Asg5) OE33 (**A**), MCF7 (**B**) and 1833-BoM (**C**) cells. (**D**–**F**) Incucytes proliferation of OE33, MCF7 and 1833 under different genotype. $N = 3$. (**G**–**N**) FACS-based quantification of the relative abundances of different cell populations in competition assays. 1833-BoM cells: 500 nM ARV-771; MCF7 cells: 200 nM ARV-771; OE33 cells: 400 nM ARV-771. $N = 3$ for each condition and timepoint (mean and SD). Source data are available online for this figure.

© The Author(s)
